# Advancing Thermoelectric Materials: A Comprehensive Review Exploring the Significance of One-Dimensional Nano Structuring

**DOI:** 10.3390/nano13132011

**Published:** 2023-07-05

**Authors:** Mustafa Majid Rashak Al-Fartoos, Anurag Roy, Tapas K. Mallick, Asif Ali Tahir

**Affiliations:** Solar Energy Research Group, Environment and Sustainability Institute, University of Exeter, Penryn Campus, Cornwall TR10 9FE, UK; ma994@exeter.ac.uk (M.M.R.A.-F.); t.k.mallick@exeter.ac.uk (T.K.M.)

**Keywords:** electrical conductivity, figure-of-merit, one dimensional, nanostructuring, materials, seebeck coefficient, thermal conductivity, thermoelectric

## Abstract

Amidst the global challenges posed by pollution, escalating energy expenses, and the imminent threat of global warming, the pursuit of sustainable energy solutions has become increasingly imperative. Thermoelectricity, a promising form of green energy, can harness waste heat and directly convert it into electricity. This technology has captivated attention for centuries due to its environmentally friendly characteristics, mechanical stability, versatility in size and substrate, and absence of moving components. Its applications span diverse domains, encompassing heat recovery, cooling, sensing, and operating at low and high temperatures. However, developing thermoelectric materials with high-performance efficiency faces obstacles such as high cost, toxicity, and reliance on rare-earth elements. To address these challenges, this comprehensive review encompasses pivotal aspects of thermoelectricity, including its historical context, fundamental operating principles, cutting-edge materials, and innovative strategies. In particular, the potential of one-dimensional nanostructuring is explored as a promising avenue for advancing thermoelectric technology. The concept of one-dimensional nanostructuring is extensively examined, encompassing various configurations and their impact on the thermoelectric properties of materials. The profound influence of one-dimensional nanostructuring on thermoelectric parameters is also thoroughly discussed. The review also provides a comprehensive overview of large-scale synthesis methods for one-dimensional thermoelectric materials, delving into the measurement of thermoelectric properties specific to such materials. Finally, the review concludes by outlining prospects and identifying potential directions for further advancements in the field.

## 1. Introduction

Climate change has caused considerably greater adverse impacts than previously anticipated by scientists a few years ago. This outcome stems from the rapid escalation in the utilization of fossil fuels to meet the surging demands of the residential, industrial, and transportation sectors. These demands have intensified in response to the rapid expansion of the global population, which surpassed 8 billion individuals in November 2022 [1]. In addition, the low energy efficiency of conventional sources has led to greater fuel consumption and a high carbon footprint. As a result, carbon dioxide emissions from fossil fuels are expected to rise by 1% worldwide in 2022 [2]. If this situation holds, humans will have produced enough CO_2_ in the atmosphere to cause global temperatures to rise by 1.5 °C above pre-industrial levels in just nine years [2]. The escalating need to mitigate CO_2_ emissions has amplified the call for alternative and sustainable energy sources. Numerous clean energy options exist, including solar, wind, geothermal, hydroelectric, and piezoelectric. Nonetheless, to effectively combat the adverse consequences of climate change, it has become imperative to harness the full spectrum of available green energy sources.

In the current era, the scientific community has directed its attention towards exploring alternative energy sources and enhancing energy conversion efficiency. Among the focus areas, thermoelectric (TE) materials have garnered significant interest. These materials are uniquely capable of directly converting heat into electricity, making them a promising candidate for clean energy applications. By harnessing waste heat from various energy generation systems, including renewable sources, TE materials offer the potential to reduce environmental pollution and enhance energy conversion efficiency. To illustrate this concept, consider the example of a conventional oil-based power generator, which typically exhibits an efficiency of only 30%. This means 70% of the chemical energy is dissipated as waste heat. However, by integrating a TE generator, the overall efficiency can be increased by approximately 2.5% by converting heat into usable electricity. Consequently, when a TE generator is attached to a fossil fuel generator, substantial annual savings of around $50,000 in fuel costs can be achieved, along with the mitigation of approximately 156 tons of CO_2_ emissions [3].

Therefore, using TE materials in energy conversion systems presents a significant opportunity to enhance sustainability and address the global energy crisis. By efficiently converting waste heat into valuable electrical energy, TE materials contribute to improving clean energy technologies and reducing greenhouse gas emissions.

TE devices made of TE materials are considered simple to operate, compact, silent, and durable, as there are no moving parts. These devices are reliable and have been used in many specialised industries, such as waste heat recovery from power plants [4] and automobiles, to reduce fuel consumption [5]; atomic batteries for power generation in the aerospace field [6]; wearable TE materials to harvest heat from the human body [7]; solar-thermoelectric generators (TEGs) applications [8]; sensors for medical applications [9]; and glazing for energy efficient buildings [10]. However, the poor efficiency of the currently available TE devices and the sustainability of state-of-the-art materials are holding back the wider usage of this technology [11].

TE technology should be capable of competing efficiently with current technologies to grow into non-niche applications [12]. The energy conversion efficiency of TE materials is mainly evaluated by a dimensionless TE figure of merit (ZT). *ZT* is defined by the equation $ZT=\frac{S^{2}\sigma }{k}T$ where *S*, σ, k, T is the Seebeck coefficient, the electrical conductivity, thermal conductivity, and absolute temperature, respectively [13]. Therefore, *ZT* is inversely proportional to the thermal conductivity and directly proportional to the Seebeck coefficient and the electrical conductivity. *ZT* of at least 4 is necessary to compete on efficiency with other thermal-to-electric conversion technologies such as organic Rankine and Kalina cycles, where the efficiency of the conversion of heat into work for these cycles range from 40% to 50% [14]. 

**Figure 1:**
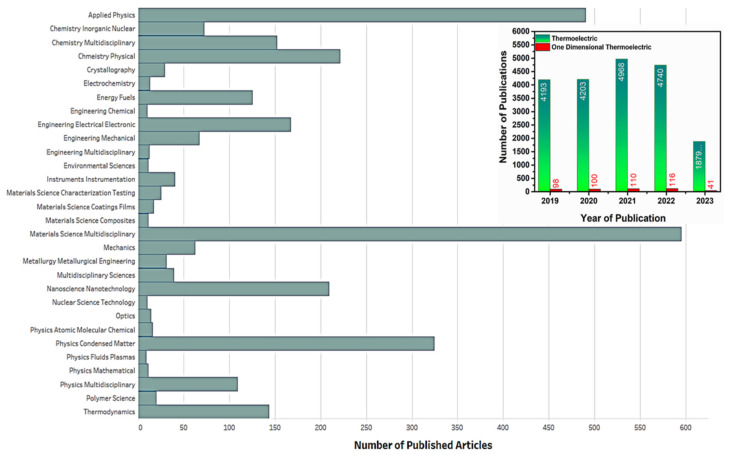
Comparison of publications on 1D TE and TE and application distribution across disciplines for 1D TE materials [15].

TE materials with high-performance efficiency are often expensive to produce, contain toxic elements, and rely on rare-earth elements. Consequently, significant research focuses on developing low-cost, high-efficiency, and environmentally friendly TE materials derived from abundant elements. One-dimensional (1D) nanostructure TE materials have gained considerable attention from researchers worldwide due to their unique properties. Despite the abundance of review articles on TE materials, few specifically concentrate on 1D TE materials, as visually depicted in Figure 1. An extensive search using the keyword “thermoelectric” in the Web of Science database has yielded an astonishing number of articles (60,439), underscoring the consistent progress and advancements within this field concerning recent technological innovations. However, when focusing on the realm of 1D TE, the overall volume of research articles diminishes considerably to a mere 1445, in contrast to the broader category of TE. 

Consequently, the primary objective of this review is to offer a more concentrated analysis of 1D TE, meticulously examining their prospects and appraising the notable innovations achieved thus far from an advanced technological standpoint. Figure 1 visually demonstrates the remarkable adaptability of 1D TE materials across various domains, with materials science and applied physics assuming principal roles as contributing disciplines. The graph illustrates the significant difference in the number of research publications dedicated to 1D TE compared to the broader field of TE. 

Thus, comprehending the significance of 1D materials in practical applications becomes paramount, necessitating further investigation. This review endeavours to shed light on this captivating field’s fundamental aspects and future possibilities. It aims to fill that gap by providing a comprehensive overview of the history, fundamental principles, and recent advancements in TE materials. It critically evaluates various materials based on their chemical structures, efficiency, and physical properties and explores potential approaches to enhance their performance. Specifically, it extensively examines the impact of 1D nanostructuring as one of the most effective methods to enhance TE materials. Various configurations of 1D nanostructured TE materials are compared and analysed regarding their influence on TE parameters. Additionally, the paper highlights key large-scale synthesis methods for 1D nanostructure TE materials and discusses characterization techniques to evaluate their performance. Overall, this comprehensive overview serves as a valuable resource for materials scientists, chemists, physicists, and engineers, particularly young researchers, assisting them in making informed decisions regarding suitable materials for their applications and strategies for developing desired material properties.

### 1.1. Historical Background

Experiments by Italian scientists (Luigi Galvani and Alessandro Volta) at the end of the 18th century were the first evidence of the direct conversion between thermal and electrical energy [16]. In (1770–1831), the German physicist Thomas Johann Seebeck noticed that when a magnetic needle is brought near a circuit of two dissimilar metals with their connections maintained at different temperatures, it deflects the field [17]. This is because the temperature difference will force the charge to flow through the conductor, and this phenomenon is known as the Seebeck effect. In 1834, a French watchmaker and part-time physicist, Jean Peltier (1785–1845), discovered an alternative to the Seebeck effect in which the flow of an electric current caused temperature differences at the junction of two dissimilar metals, known as the Peltier effect [18]. William Thomson (1824–1907), often referred to as Lord Kelvin, was the first to link these two fundamental phenomena in 1851. The Seebeck coefficient multiplied by absolute temperature equals the Peltier coefficient; in his thermodynamic derivation, Thomson predicted that there would be a third TE effect, now known as the Thomson effect [19,20]. 

Edmund Altenkirch (1880–1953) proposed a theory for calculating the efficiency of TEGs in 1909 and the performance of a cooler in 1911, demonstrating that a suitable material should have a high Seebeck coefficient, high electrical conductivity, and low thermal conductivity. This relationship was later developed by Abram Fedorovich Ioffe (1880–1960) into the “figure of merit” (ZT) [19,21]. Maria Telkes (1900–1995) successfully constructed the first (solar) TE power generator in 1947 and the TE refrigerator in 1953 using semiconductor thermoelectricity principles. The average efficiency of 5% for Telkes’s generators was reached [22]. By the 1950s, cooling temperatures below 0 °C had been achieved by H. Julian Goldsmid and R. W. Douglas, enabling the development of several successful companies [19,20]. 

Glen Slack invented the “Phonon Glass Electron Crystal” concept in TE in the 1990s, separating electrical and thermal conductivities in the same material. This concept is based on the idea that semiconductors can be designed such that electron transmission should be as efficient as a crystalline conductor while phonon should be widely blocked in the lattice, as in a glass. This theory changed the search methods for the design of TE materials [23,24,25,26,27]. In 1993, L. D. Hicks and M. S. Dresselhaus performed theoretical research and found that nanostructures could significantly impact the TE materials’ properties [28,29]. 

**Figure 2:**
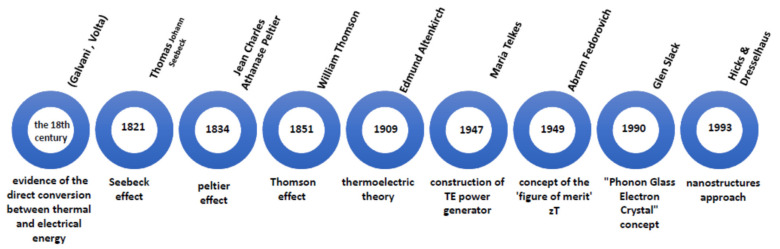
The key achievements in the evolution of the TE sector.

Figure 2 illustrates the key milestones in the historical advancement of TE materials. Despite considerable endeavours to enhance TE materials and improve the efficiency of TE devices through strategies such as resonant level doping or the integration of complex materials, it can be inferred that the progress in TE materials has been relatively modest.

### 1.2. Basics of Thermoelectric Materials

**Figure 3:**
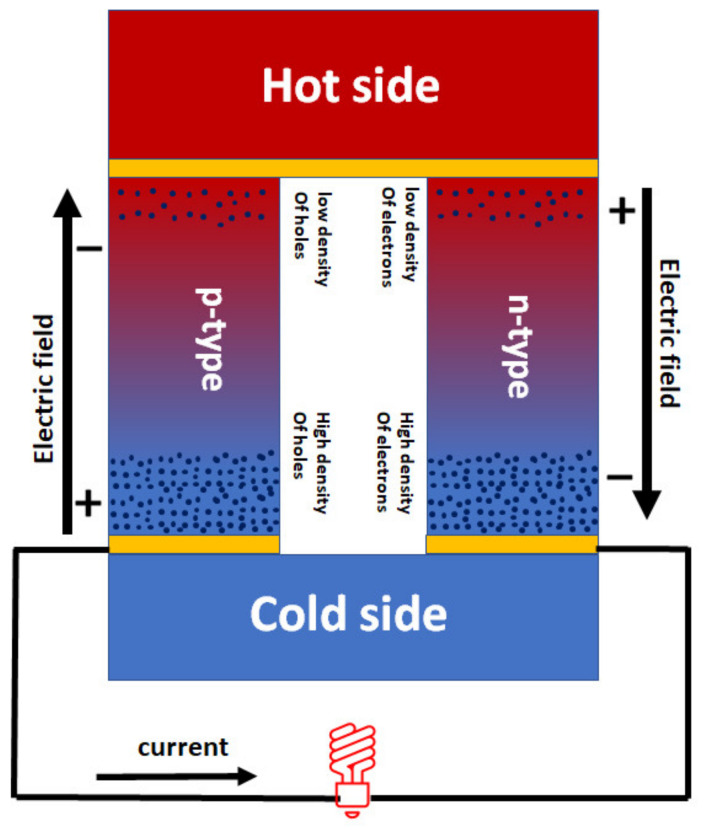
Schematic illustrating the working principle of TE materials.

TE materials working principle is built on the Seebeck effect. The Seebeck effect occurs when a temperature gradient is applied to the junction of metals, causing the charge carriers to move from the hot side to the cold side. This results in a charge concentration on the cold side and an induced potential that generates current when connected to circuits [30]. Generally, TE devices are made up of flat array junctions of p-type (most charges are positive) and n-type (most charges are negative) that are connected electrically in series and thermally in parallel, as shown in Figure 3 [18]. P-type materials experience a positive voltage concerning the hot side of a temperature gradient, while n-type materials experience a negative voltage. The difference in voltage between these voltages will cause current to flow from the positive voltage of the p-type material to the negative voltage of the n-type material.

*ZT* determines the efficiency of the energy conversion at a given temperature, which the following Equation (1) can express:

(1) $$ZT=\frac{S^{2}\sigma }{k}T$$

where S, σ, k, T are the Seebeck coefficient, the electrical conductivity, thermal conductivity, and absolute temperature, respectively [13]. Furthermore, *ZT* influences the TE device’s energy conversion efficiency (η). The electrical power output (W) ratio to thermal power provided (QH) determines the power production efficiency. This is indicated by the following equation (Equation (2)):

(2) $$\eta =W/\mathrm{QH}=\frac{T_{h}-T_{c}}{T_{h}}\times \frac{\sqrt{1+ZT-1}}{\sqrt{1+ZT}+\frac{T_{c}}{T_{h}}}$$

where (*T*_h_) is the temperature on the hot side, and (*T*_c_) is the temperature on the cold side [31]. The preceding equation demonstrates that *ZT* and efficiency are proportional. To achieve high conversion efficiency, TE materials should have a high *ZT*. High *ZT* requires high Seebeck, high electrical, and low thermal conductivity. 

The Seebeck coefficient (S) is primarily defined by the number of valence electrons that gain mobility due to thermal energy input, and it can be expressed using the following equation (Equation (3)):

(3) S=ΔV/ΔT

*S* is the Seebeck coefficient measured by μV/K, and *V* is the electrical voltage [32]. Seebeck coefficients are affected by several variables, including temperature and the material’s chemical composition at a specific temperature [33]. The Seebeck coefficient could be expressed by the Pisarenko relationship for a detergent semiconductor as in the equation below (Equation (4)):

(4) S=8π2KB2T3eh2m*π3n23

where $k_{B}$, e, m*, h, and n represent the Boltzmann constant, the carrier charge, the DOS effective mass of the charge carrier, Planck’s constant, and the carrier concentration, respectively [34]. From Equation (4), it can be concluded that an increase in m* will increase Seebeck. 

Equation (5) below expresses electrical conductivity in the term of electrons.

**Figure 4:**
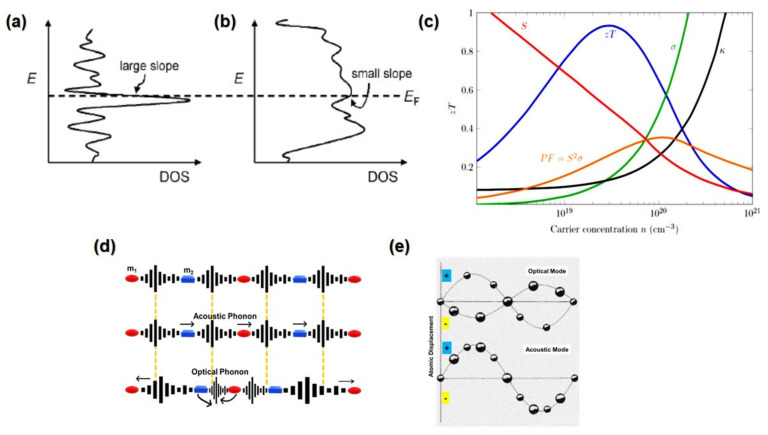
The theoretical density of states (DOS) with (**a**) Large slope (**b**) Slight slope “Reproduced with permission from [36], WILEY-VCH, 2009”. (**c**) The effect of the carrier concentration on the Seebeck, electrical conductivity, thermal conductivity, TE power factor and *ZT* based on data from ref. [37]. (**d**) Acoustic and optical phonons in a 1D chain of atoms. Atoms with masses m1 (red) and m2 (blue) are arranged in an alternating configuration, while their displacements are represented by arrows [38]. (**e**) Different types of atomic displacement modes [39].

(5) σ=neμ

whereas *μ* is the carrier mobility [31]. The equation above indicates that electrical conductivity can be enhanced by increasing the concentration and mobility of electrons. However, from Equations (4) and (5), it can be concluded that increasing the number of carriers will increase electrical conductivity but decrease the Seebeck coefficient. Thus, to achieve a high value for Seebeck, TE materials must tune an appropriate n (number of n-type carriers) or p (number of p-type carriers). Generally, the major carriers in semiconductors are the holes in p-type and the electrons in n-type, while the minor carriers are the electrons in p-type semiconductors and the holes in n-type semiconductors. The relationships between n or p, σ, *S*, and *T* can also be described by Boltzmann’s transport theory [35]. This theory comprehensively explains the thermopower described by the Mott equation (Equation (6)).

(6) $$S={\left.\frac{\pi^{2}K_{B}{}^{2}T}{3e}\frac{d\ln\sigma \left(E\right)}{dE}\right]}_{E=E_{f}}$$

where $k_{B}$ is the Boltzmann constant, E is the electron energy, $E_{f}$ is the electron energy at the Fermi level, and$\;\sigma \left(E\right)$ is the electronic conductivity specified in terms of the Fermi energy ($E_{f}$) or band filling [35], which also can be expressed as below (Equations (7) and (8))

(7) $$\sigma \left(Ε\right)=n\left(E\right)e\mu \left(E\right)$$

with carrier density

(8) $$n\left(Ε\right)=g\left(E\right)f\left(E\right)$$

whereas $g\left(E\right)$ and $f\left(E\right)\;$are the density of states (DOS) and the Fermi function, respectively. DOS is the number of different states available at a given energy level. If electronic scattering has no energy dependency, there will be proportionality between $\sigma \left(E\right)$ and the density of states (DOS) at E. in Figure 4a,b; there are two hypothetical electronic DOS diagrams: one where the DOS fluctuates significantly around $E_{f}$ and one where it does not [36]. According to Equation (4), the system in Figure 4a, which has a rapidly varying DOS, is predicted to have a higher TE power.

The power factor (*PF)* can be used to express the relationship between electrical conductivity and the Seebeck coefficient, as shown in Equation (9):

(9) $$PF=S^{2}\sigma$$

*ZT* is directly proportional to the power factor, and the power factor can be improved by tuning a certain amount of carrier concentration [40]. Figure 4c illustrates the effects of carrier concentration on TE parameters. Increasing the carrier concentration will increase electric and thermal conductivity, but the Seebeck will decrease sharply. The optimal value of Seebeck and electrical conductivity can be found in heavily doped semiconductors when the carrier concentration ranges between 10^19^ and 10^21^ [41]. The power factor will increase gradually with increasing carrier concentration and reach a peak before dropping. *ZT* will increase sharply and reach a peak by increasing the carrier concentration, then drop dramatically while increasing the carrier concentration. It can be noticed that the peak of *ZT* occurs when the thermal conductivity is low.

In materials, thermal conductivity (*k*) is a combination of the phonon component of lattice vibrations (𝑘*_l_*) and the electronic component (𝑘*_e_*) and can be expressed by the following equation (Equation (10)):

(10) $$k=D_{T}C_{P}\rho =k_{e}+k_{l}$$

where $D_{T}$ is the thermal diffusivity, $C_{P}$ is the specific heat, and ρ is the mass density [42]. The Wiedemann–Franz relation also shows that high thermal conductivity in degenerate semiconductors and metals is linked to electronic properties as it is directly related to electrical conductivity at a given temperature [43], as described in Equation (11)

(11) $$k_{e}=L\sigma Τ=ne\mu LT$$

where *L* is the Lorenz number (2.45 × 10^−8^ WΩk^−2^) related to a free electron. Because 𝑘*_e_* is related to the Lorenz factor, which changes with carrier concentration and transport mechanism, it may not be changed significantly, except for low-carrier concentration materials, where the factor can be reduced by up to a fifth from its free-electron value [41]. Moreover, it can conclude from the equations above that thermal conductivity will increase when electrical conductivity increases in a TE material.

The phonon component of lattice vibrations (𝑘*_l_*) can be expressed as Equation (12):

(12) $$k_{l}=\frac{1}{3}c\nu l\;$$

where c represents the heat capacity per unit volume, ν corresponds to the sound velocity, and l is the phonon mean free path. It is possible to decrease the lattice thermal conductivity by increasing phonon scattering, which can be accomplished by many methods, such as nanostructuring, alloying, complex structures, etc. [41].

Thermal energy is carried in solids by two types of phonons: acoustic and optical phonons. Acoustic phonons are coherent atom displacements to propagation, whereas optical phonons are incoherent movements of two neighbouring atoms in opposite directions, as shown in Figure 4d,e [38,39,44]. These phonons can move in parallel, longitudinal, perpendicular, or transverse directions. The optical phonon superiority is more substantial in places with higher temperatures, whereas the acoustic phonon superiority is more significant in locations with lower temperatures. Furthermore, acoustic phonons have a longer mean free path than optical phonons [45]. Acoustic branch group velocities are also larger than optical branches in longitudinal and transverse modes [44].

At low temperatures, boundary scattering predominates. When temperature increases, it will reduce the wavelength of the dominant phonons that transport heat, resulting in phonon diffusion caused by defects, displacements, or another process. Impurity scattering is typically apparent at the thermal conductivity peak, whereas phonon-phonon scattering occurs at higher temperatures [46]. That leads to the understanding that an increase in temperature will reduce the thermal conductivity of the lattice [47].

Based on the observations mentioned above, it can be deduced that the TE parameters exhibit interdependence. This mutual reliance poses a significant challenge for improving individual parameters without affecting others. Nevertheless, various strategies exist to overcome this interdependency, which will be thoroughly discussed in the subsequent sections of this comprehensive review.

## 2. State-of-the-Art TE Materials

**Figure 5:**
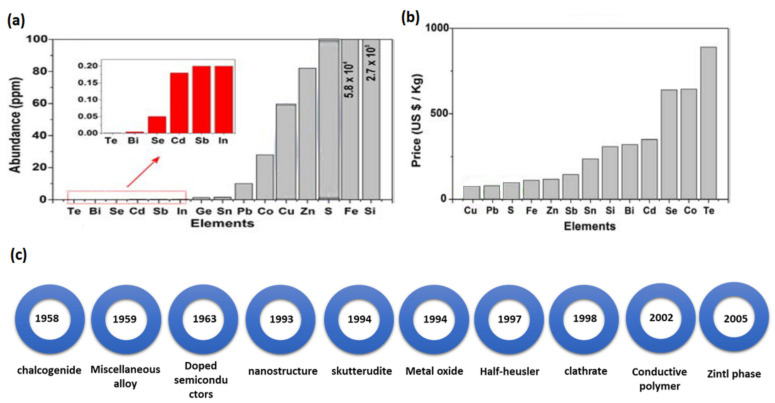
(**a**) The abundance of various elements on the earth’s crust. (**b**) Price of the common TE elements “Reproduced with permission from [49], WILEY-VCH, 2019”. (**c**) State of the art in TE materials discovery Cu and S (green) are the prevailing elements in terms of abundance and cost-effectiveness for the development of TE materials.

TE material should have some requirements to be efficient, such as possessing high electrical conductivity, low thermal conductivity, and high Seebeck. Commercial TE materials have been mostly based on alloys with *ZT* > 1, such as Bi_2_Te_3_, PbTe, and SiGe, for many years. However, the use of these traditional TE materials is limited because they are toxic, rare, and expensive. For example, the main element of traditional TE materials is scarce, with only 0.001 ppm, as shown in Figure 5a [48]. While lead is one of the most critical elements in traditional TE, it is highly toxic. In addition, Te and Ge are considered expensive compared with other materials, as shown in Figure 5b [49]. As a result, researchers have been making great efforts to invent or develop the performance of materials that are cheap, abundant, and environmentally friendly, such as oxides, Zintl phase, carbon-based compounds, polymers/organic materials, Half-Heusler compounds, sulphides, and perovskite. Figure 5c illustrates state of the art in TE materials discovery [50]. This section will discuss the traditional and modern TE materials.

### 2.1. Bismuth Telluride

One of the traditional, effective TE materials in low temperatures is Bismuth telluride (Bi_2_Te_3_), part of the V–VI group of chalcogenide semiconductors [51]. Bi_2_Te_3_ shows good thermal, mechanical, and chemical stability. The Peltier effect (thermal cooling) has been seen in p-type Bi_2_Te_3_ linked with n-type samples since the early 1960s and has been commercialised [41]. This TE materials group is superior for several reasons. They are very anisotropic, with strong electrical conductivity, higher thermopower, an excellent Seebeck coefficient, and a lower thermal conductivity in the direction perpendicular to the c-direction [41].

**Figure 6:**
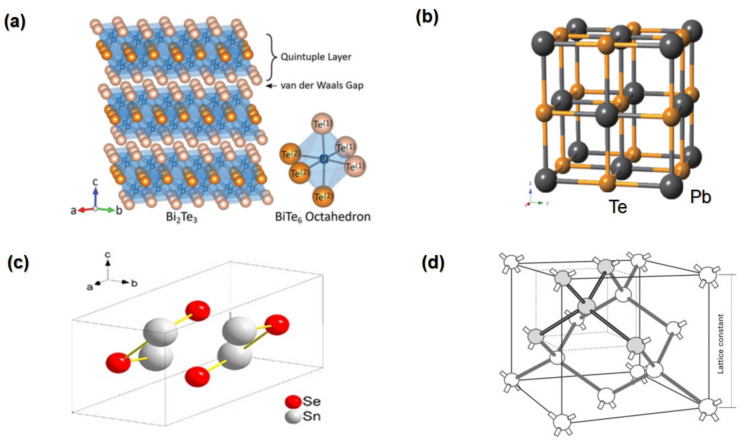
(**a**) Atomic structures of a bulk unit cell of Bi_2_Te_3_ “Reproduced with permission from [51], WILEY-VCH, 2019”. (**b**) Typical PbTe cell displayed with simple cubic structure “Reproduced with permission from [61], American Physical Society, 2012”. (**c**) The unit cell of α-SnSe crystal structure “Reproduced with permission from [62], Elsevier, 2018”. (**d**) Crystal structure of the SiGe “Reproduced with permission from [63], Woodhead, 2011”.

Figure 6a shows the atomic arrangement in A_2_B_3_ (A = Bi, Sb; B = Se, Te, S) compounds. Bulk Bi_2_Te_3_ has a rhombohedral crystal structure with the space group R3m. Its structure comprises a quintuple of five atomic layers, Te-Bi-Te-Bi-Te, arranged in the Z-direction [52]. Weak van der Waals forces separate the quintuple layers from one another [52]. Due to these weak bonds between subsequent quintuples, this compound has a layered structure, and the crystal can be readily fractured in this direction [41].

Several ways to increase Bi_2_Te_3_ TE efficiency include enhancing electronic transport characteristics, modifying carrier concentration via doping, alloying, and band structure engineering, and lowering phonon conductivity via structural dimension reduction [41].

One of the most effective methods is nanostructuring. For example, p-type bismuth telluride has a high *ZT* = 1.80 at 316 K in nanostructuring [53], 21% higher than the bulk of *ZT* = 1.41 at 300 K [52]. Moreover, the Seebeck coefficient of the p-type Bi_0.46_Te_0.54_ is 260 μVK^−1^ at 300 K, which is 60% greater than that of the bulk crystal of the Bi_0.46_Te_0.54_ [54].

### 2.2. Lead Telluride

Lead telluride (PbTe) is a semiconductor chalcogenide with a narrow bandgap TE material [41]. Pb (S, Se, Te) compounds were among the first TE materials found. As a rock salt crystal, it has octahedral molecular geometry as its structure, and its lattice dynamics are very anharmonic, as shown in Figure 6b [41]. At low temperatures, it is highly brittle and has easy cleavage. As the temperature rises, the cleavage becomes minor or vanishes [55]. PbTe is very interesting due to its unique electronic properties and possible applications in infrared detectors, light-emitting devices, infrared lasers, thermophotovoltaics, and TE [41].

PbTe is often a p-type TE material used in moderate temperatures, although n-type conduction has also been seen due to the abundance of Pb in PbTe [41]. P-Type conduction can be achieved by doping acceptors (Li, Na, K, Rb, Cs, Tl, etc.) to fine-tune the carrier concentration [55]. For instance, a high *ZT* was achieved by doping Na with PbTe by increasing the Seebeck. The *ZT* of p-type Na-doped PbTe reached 1.4 at 750 K [6]. Alternatively, n-type conduction can be obtained by doping Ga, In, La, Sb, Al, and Bi [55]. For instance, the *ZT* value of Al-doped PbSe approaches 1.3 at 850 k due to the increased Seebeck caused by the resonant state in the conduction band [56].

The nanostructure method is widely used to enhance the transport properties of PbTe TE materials. As an example of the nanostructure method, the nano inclusions considerably increase the phonon scattering and decrease the thermal conductivity of the lattice. For example, ultra-low lattice thermal conductivity can be obtained from 3% Na doped (PbTe)_0.8_(PbS)_0.2_ prepared by Spark Plasma Sintered due to the in situ formed sulphur-rich nano-precipitates. With tuning carrier concentration by Na, a maximum *ZT* value of 2.3 was achieved at 923 K [57]. The highest *ZT* value recorded for PbTe was around 2.5 at 923 K was achieved in the PbTe-SrTe system by reducing the grain size and tuning the carrier concentration [58]. Ultra-thin PbTe NWs (12 nm in diameter) show a high reduction in thermal conductivity compared with bulk [59]. Sharma et al. (2021) review recent developments in PbTe TE [60].

### 2.3. Tin Selenide

Tin selenide (SnSe) is well-known for possessing semiconducting properties used in various products, including solar cells and phase-change memory alloys [64]. SnSe is a highly stable low-toxicity compound composed of abundant earth elements. Because of its complex, layered structure, the SnSe crystal shows an innately extremely low thermal conductivity. In recent years, SnSe has been identified as one of the most promising TE materials at medium temperatures.

SnSe shows the space group Pnma at room temperature, whereas at higher temperatures, there is a phase transformation from the Pnma to the Cmcm space group [65]. Figure 6c shows a unit cell of SnSe with eight atoms [62]. Strong heteropolar bonds connect the Sn and Se atoms to create the crystalline layers, which comprise two planes of zigzag Sn−Se type chains [66]. Each Sn atom is connected to three Se atoms, and each Se atom is likewise bound to three Sn atoms. The neighbouring layers are primarily connected by van der Waals forces and long-range electrostatic attractions [62].

*ZT* varies along the three crystallographic axes due to differences in electrical conductivity, with the b and c axes having higher conductivity than the a axis due to increased carrier mobility. SnSe single crystals showed a high *ZT* value of 2.6 along the b axis, 2.3 along the c axis, and 0.8 along the a axis at 923 K [65]. In contrast to the isotropic behaviour of the Seebeck coefficient, the lattice thermal conductivity decreases dramatically in the high-temperature region, with the lowest value along the a axis due to high anharmonicity [65].

The TE performance of the SnSe could be enhanced by increasing the carrier concentration through doping. A high *ZT* value of 2.2 has been achieved at 733 K along the b axis for Bi-doped SnSe single crystals [67]. Moreover, acceptor doping in SnSe single crystals could improve the TE properties of SnSe in the Pnma phase. This is because the electronic transport properties improve as the Fermi level shifts, the valence band edge is flattened, and the number of carriers increases. For example, a *ZT* value of 1.17 in the temperature range 300 K–800 K has been achieved by Na doped SnSe single crystals with the peak *ZT >* 2 at 800 K along the b axis [68]. The *ZT* values for doping some elements such as: Ge, Pb, Y, Cd, and As has been theoretically calculated [69].

Polycrystalline SnSe has lower thermal conductivity than the single crystal due the high phonon scattering at grain boundaries. However, polycrystalline SnSe reduces carrier mobility at high temperatures, reducing the electrical conductivity [68]. Thus, the value of *ZT* for a single crystal is higher than for polycrystalline SnSe.

Various efforts have been made to enhance the TE properties of polycrystalline SnSe. For example, the highly textured structure of SnSe crystals effectively increases the electrical conductivity, which leads to a *ZT* value of 0.92 at 873 K in p-type polycrystalline SnSe [70]. Furthermore, Na doping polycrystalline SnSe could enhance the *ZT* value as the carrier concentration increases and thermal conductivity is reduced. For example, Chere et al. achieved a *ZT* value of 0.8 at 773 K by Na doping polycrystalline SnSe [71]. Moreover, a *ZT* value 1 at 773 K may be obtained by alloying the polycrystalline SnSe with 10% SnS [71].

The inclusion of the PbSe phase to the SnSe compounds can also increase electrical conductivity and power factor. In addition, a point defect engineering approach Field could improve TE performance in the n-type polycrystalline SnSe [72]. Zhou et al. (2021) produced hole-doped tin selenide (SnSe) polycrystalline TE samples and obtained a *ZT* value of 3.1 at 783 K, 41% more than single-crystal SnSe samples. This enhancement is attributed to the high reduction of the thermal conductivity of 0.07 W m^−1^ K^−1^ due to phonon scattering at the polycrystalline SnSe interface [73]. Nanostructure could be an excellent approach to increase the efficiency of SnSe. For example, SnSe NWs achieve *ZT* of 0.156 at near room temperature. This relatively high *ZT* is attributed to the low thermal conductivity of 0.55 Wm^−1^ K^−1^ [74]. Aspan et al. (2021) provide an overview of SnSe and its applications in TE [75].

### 2.4. Silicon-Germanium System

The silicon and germanium system is responsible for most TE power generation, particularly for power generation applications at high temperatures. It is an eco-friendly material with high mechanical, thermal, and chemical stability, while Ge is a rare earth element. Although Si and Ge have high power factors, their high heat conductivity disqualifies them as TE materials [55]. In the 1960s, it was discovered that alloying Si with Ge could significantly reduce the lattice thermal conductivity while retaining reasonably high electron mobility [55].

The operating temperature range for SiGe-based TE materials is 600–1000 C, with a *ZT* of 1.3 for n-type [76] and 0.95 for p-type [77] at 900 C, respectively. To control the carrier concentration in Si-Ge alloys, elements from group V, such as P and As, are usually utilised as donor dopants, whereas B and Ga are used as acceptor dopants [55].

Figure 6d shows the crystal structure of SiGe, where SiGe alloys have a cubic diamond lattice (space group Fd3m) comprising two face-centred cubic primitive lattices that cross over each other [41]. Due to their cubic structure, SiGe alloys have good electrical and TE performance that can be improved by lowering the thermal conductivity of their lattices [78]. When Nozariasbmarz and his colleagues (2016) add silicide nanoinclusions into the SiGe alloy, the power factor is enhanced, and the thermal conductivity is reduced, resulting in an increase in the *ZT* value of 1.3 in the Si_0.88_Ge_0.12_-Mg_2_Si nanocomposite at 1200 K [79].

Nanostructure is an effective method for enhancing TE performance by reducing lattice thermal conductivity. For example, compared to the 7–8 Wm^−1^K^−1^ recorded in bulk at 300 K, the thermal conductivity of SiGe NWs is only around 1–2 Wm^−1^K^−1^ [80].

Nanocomposites are also useful for enhancing the TE characteristics of bulk SiGe. Compound nanoparticles as second-phase candidates will lower the lattice thermal conductivity without significantly reducing electrical properties. Adding metallic Yttrium silicide nanoparticles increased the *ZT* value of P-type SiGe alloys to 1.81, resulting from the thermal conductivity reduction due to the creation of coherent states in the SiGe matrix and the reduction of grain size [81]. Basu et al. (2021) summarised recent information about the TE Si-Ge alloys [82].

### 2.5. Skutterudites

**Figure 7:**
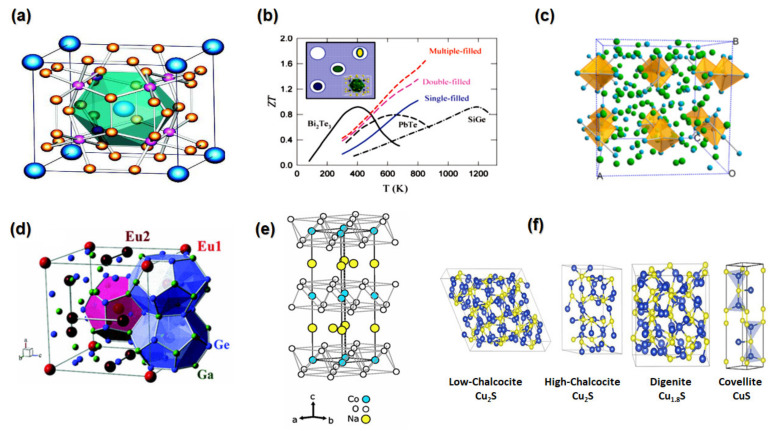
(**a**) Crystal structure of CoSb_3_, where small pink is Co, small yellow is Sb atoms, and the large blue sphere is filler “Reproduced with permission from [131], American Physical Society, 2011”. (**b**) Temperature-dependent *ZT* value of single, double, and multiple filled skutterudites “Reproduced with permission from [87], American Chemical Society, 2011”. (**c**) The crystal structure of Yb_14_MnSb_11_ along the c axis. Yb, Mn, and Sb atoms are indicated by green, orange, and blue spheres, respectively. “Reproduced with permission from [93], American Chemical Society, 2015”. (**d**) Unit cell of the clathrate type-I Eu_8_Ga_16_Ge_30_ “Reproduced with permission from [100], American Physical Society, 2013”. (**e**) Crystal structure of Na_x_CoO_2_ “Reproduced with permission from [132], American Physical Society 2006”. (**f**) The crystal structure of different Cu_2−x_S “Reproduced with permission from [124], Elsevier, 2021”.

These are often composites of metal and pnictogen elements in MX_3_, where Co, Fe, Rh, and Ir represent M and P, As, and Sb represent X, respectively [18,41,55]. Figure 7a shows the crystal structure of skutterudites, which is a compound that has 32 atoms, eight cubic sublattices, and is formed of metal elements; six of the cubes are filled with pnictogen square planar rings and create an octahedral structure with the metal elements [18,41,55]. This category of chemicals has a cubic space group Im3 crystal structure [83].

Due to the crystalline structure of skutterudite compounds, skutterudites show typical PGEC properties [41]. Specifically, filled skutterudites at medium temperatures were identified as one of the best TE materials for power generation [41]. Different chemical nature A (rare-earth, alkaline-earth, or alkali metals) could fill the binary skutterudite to create the ternary skutterudite A_2_M_8_X_24_ (half of the unit cell AM_4_X_12_ that reduces thermal conductivity by providing resonant phonon scattering) [18,41,55]. Due to the combination of large unit cells and heavy atomic weights, their complicated lattice structure is the primary cause for their low heat conductivity [41].

There are two effective methods to improve the *ZT*: either by alloying, which involves either substituting host atoms or introducing defects into the lattice cage (a process called “filling”), or by reducing system dimensions [41]. Partially filled skutterudites with different filler types exhibit distinctive structural properties, optimising electrical and thermal transports independently and increasing ZT values in single, double, and multiple-filled configurations, as shown in Figure 7b. Of all the skutterudites, CoSb_3_ is the most researched due to its environmentally friendly composition, excellent mobility, low electrical resistivity, large atomic masses, and good Seebeck coefficients [84]. However, CoSb_3_ cannot reach a high *ZT* due to its high lattice thermal conductivity (larger than 10 Wm^−1^K^−1^ at room temperature) [26,55].

The thermal conductivity of the lattice could be significantly decreased by filling the structural gaps with suitable filler elements [85]. For example, filling CoSb_3_ with Fe significantly decreases thermal conductivity, causing p-type La(Fe,Co)_4_Sb_12_ to achieve a high *ZT* value of 0.9, greater than 0.5 for CoSb_3_ [26]. The skutterudite filled with abundant earth elements, such as ZnO nano inclusions, improved the *ZT* up to 36% at 300 K [86]. *ZT* can be enhanced by further reducing thermal conductivity by adding two or more filler atoms with a wide range of vibrational frequencies. For example, multi-filled skutterudite Ba_0.08_La_0.05_Yb_0.04_Co_4_Sb_12_ has a *ZT* of 1.7 at 850 K [87].

### 2.6. Zintl Phase

Zintl phase is a new group of materials with a promising *ZT* [18,41,55]. It is a polar intermetallic material with high TE efficiency due to its PGEC characteristics and has attracted researchers’ attention [41,88]. Zintl phase has a complex structure because it combines ionic and covalent bonds. This structure provides higher charge mobility than non-ionic material because of electron transfer from ionic cations to covalently bonded anions [49]. The Zintl phase can be easily modified structurally and chemically, allowing for substantial changes in transport properties [18,41,55,89].

Most Zintl compounds have high electrical conductivity, low thermal conductivity, and high thermal stability at high temperatures [41]. The very low thermal conductivity of Zintl compounds is caused by low-velocity optical phonons, which are mostly related to their complex structures [90].

The best-known and most promising compound in this group is Yb_14_MnSb_11_; it possesses a very low lattice thermal conductivity of (0.4 Wm^−1^K^−1^ at 300K) [91] and has been shown to have a high *ZT* of 1.3 at 1223 K [92]. The structure of Yb_14_MnSb_11_ features tetragonal symmetry and an I41/acd space group; each unit cell has eight formula units and 208 total atoms, as shown in Figure 7c [55,93].

The electrical conductivity can be enhanced by element doping and tuning appropriate carrier concentration. For example, *ZT* of 1.3 at 1223 K is obtained for Yb_14_Mn_0.2_Al_0.8_Sb_11_ by optimising carrier concentration and substituting Al^+3^ for the Mn^+2^ more than double that of the state-of-the-art Si_0.8_Ge_0.2_ flown by NASA. Moreover, the thermal conductivity of Yb_5_Al_2_Sb_6_ could be reduced by substituting only 17% of the Yb with Sr cations in Sr_0.85_Yb_4.15_Al_2_Sb_6_ [90]. Furthermore, extensive defects have been detected in the layered Zintl compound SrZnSb_2_, and it has been demonstrated that they dramatically decrease lattice thermal conductivity near room temperature to 30% [94].

Despite nanostructures’ efficacy in decreasing thermal conductivity in other materials, relatively few studies have been conducted on nanostructuring Zintl compounds. For example, nanostructuring bulk Mg_3_Sb_2_ and Mg_3_Sb_1.8_Bi_0.2_ produces *ZT* values of 0.4 and 0.94 at 773 K; these are 54% and 56% higher, respectively, than their bulk equivalents. These materials have a greater *ZT* because phonon scattering at the many grain boundaries of nanostructured materials results in a significant decrease in their thermal conductivity [95].

In recent years, Mg_3_Sb_2-x_Bi_x_ compounds have become one of the most extensively investigated TE materials due to their abundant, affordable component elements, easy synthesis, high melting point, and remarkable mechanical stability [96]. A high *ZT* value of 1.5 in the Mg_3.2_Sb_1.5_Bi_0.49_Te_0.01_ Zintl phase has been achieved by increasing the grain size from 1.0 to 7.8 µm [97]. Mg_3_Sb_2−*x*_Bi*_x_* TE materials can efficiently work at room temperature. For example, Pan et al. (2020) synthesised n-type single crystal Mg_3_Bi_1.25_Sb_0.75_ and achieved a *ZT* of 0.82 at 315 K. Detailed information on the recent progress on TE Zintl phases can be found in ref. [98].

### 2.7. Clathrates

Clathrates are single-phased solids containing two individual components (the guest and the host) connected through unconventional chemical bonding by enclosing one molecule type entirely within a structure generated by the other. Clathrates are a promising TE material at high-temperature applications as they can achieve “phonon-glass electron-crystal”. Powell is the first to adopt “clathrate”, and it is named after the Latin word “clathratusclathrates” to refer to compounds with a caged structure [41,55].

Clathrates (type I) are expressed by the equation A_x_B_y_C_46−y_, where B is group III and C, are group IV atoms, together creating the framework in which “guest” atoms A (alkali or alkaline-earth metal) are held in closed voids or cages and enclosed in the polyhedra separately and are positioned facing each other [41,55]. The entire system, the cage and its molecules is considered a single cell [99]. This class of compounds has space a large void and unit cell with high symmetry, as shown in Figure 7d [100]. Guest atoms or molecules are physically trapped in the enormous voids of the lattice, which also maintains the frame structure and may not be stoichiometric, and this interaction will influence clathrate performance [55].

Clathrates have poor lattice thermal conductivity due to the intense vibrating of the guest atoms, which scatters phonons substantially [55,101]. Clathrates are categorised into several types, including type I, II,II, III, and IV, based on the number and arrangement of cages and the size of voids in their unit cells. The structure and physical properties of type I and type II clathrates have been extensively studied. However, there has been increasing research on type III and type VIII clathrates in recent years.

Si-, Ge-, and Sn-based clathrates (A_x_B_y_E_z_, where E=Si, Ge, Sn) have attracted significant attention as TE materials within the clathrate family. The enhancement of TE characteristics in semiconducting type I clathrates is commonly achieved by substituting foreign atoms and doping. Shi et al. (2010) demonstrated that introducing ionised impurities into the framework of clathrates can influence the local electrical potential field, leading to an increase in the power factor. Notably, they achieved a *ZT* of 1.2 at 1000 K for the polycrystalline bulk of Ni-doped type I clathrates Ba_8_Ni_2.97_Ga_3.94_Ge_38.91_ [102].

The TE characteristics of germanium-based clathrates vary depending on the synthesis technique due to differences in the Ga/Ge molar ratio. The Ga/Ge ratio affects the Seebeck coefficient: unfavourable values for x = 12–16 and favourable values for x = 17–20 [103]. For example, Saramat et al. (2006) reported that, at 900K, the *ZT* value of Ba_8_Ga_16_Ge_30_ produced by the Czochralski technique is 1.35 and reached approximately 1.63 at 1100 k. Recently, Shen et al. (2022) synthesised Type-VIII single-crystalline Sm_0.75_Ba_6.25_Ga_16_Sn_30_ Clathrate using the Sn-flux method. The result showed a high Seebeck coefficient and electrical conductivity at 300–600 K, leading to a ZT peak 1.31 at 457 K [104]. Detailed information about the clathrate and its application in TE can be found in [105].

### 2.8. Oxides

The interest in oxides as TE materials grew only in the late 1990s when Erasaki et al. introduced more complex oxides to the field [106]. In 2001, the announcement of a high *ZT* greater than 1.0 in Na_x_CoO_2_ sparked intense research on oxide TE [107]. These oxides were produced from the elements Na, Co, and O, with a formula of Na_x_CoO_2_ [108]. Oxide TE materials provide several benefits, including good thermal stability at high temperatures, high corrosion resistance, low cost, environmentally friendly, ease of synthesis, and exhibit electrical conductivity almost similar to metal, and good Seebeck coefficient [55].

A key idea in layered oxide TE materials is the “block module”, which allows electrical and thermal transports to be separately tuned. Na_x_CoO_2_ contains two distinct sorts of layers. The first is the incomplete Na_X_ layer, and the second is the conductive CoO_2_ layer, as shown in Figure 7e [55]. The increasing Na content will enhance the Seebeck coefficient [109]. Other p-type oxides that have been intensely examined include Ca_x_CoO_2_, Ca_3_Co_4_O_9_, Sr_x_CoO_2_, and others. Among all the p-type oxides, Ca_3_Co_4_O_9_ seems the best choice for TE applications as the Na in NaCo_2_O_4_ and the Bi in Bi_2_Sr_3_Co_2_O_9_ are very volatile [110]. CaMnO_3_ and SrTiO_3_ perovskite oxides have also received interest as possible n-type TE materials. At 1073 K, the *ZT* value of La-doped SrTiO_3_ is 0.27 [111], whereas La and Dy co-doping increased the *ZT* to 0.36 at 1045 K [112].

An interesting study has shown that Sb-doped ZnO micro/nanobelt as a TE nanogenerator produces an output voltage of 10 mV, an output current of 194 nA, and an output power of approximately 1.94 nW at a temperature gradient of 30 K [113]. Also, researchers utilised Al doping in ZnO to increase its TE efficiency [114]. Moreover, ZnO is one example of interesting oxide TE material.

Copper oxide powders and pastes were recently reported with Seebeck coefficients of 650 μVK^−1^ [115]. In addition, the TE properties of copper oxides were successfully modified by combining them with other materials, such as graphite powder, to form composites. The main disadvantage of this composite was its high thermal conductivity [116]. Among the oxide compounds for which high-throughput predictions were produced, SnO was found as a viable TE candidate with good electrical and thermal properties [117].

Nanostructuring is one of the most effective methods to enhance the oxides’ TE performance. For example, when Singh et al. (2021) synthesised n-type nanocomposite (la_0.7_Sr_0.3_MnO_3_)_0.5_. (NiO)_0.5_ by sol-gel method, the *ZT* increased by ∼285%. This triable increase came from nanostructuring and composite of two materials with different electronic structures [118]. Detailed information about the oxide TE material can be found in [119].

### 2.9. Metal Sulphides

Metal sulphides are an earth-abundant material group that has interesting electrical properties. Among all interesting metal sulphide TE materials, copper sulphides have been intensively investigated for TE applications because they are earth-abundant, cheap, low-toxicity, easy to synthesise, and high-performance TE materials [49]. Chalcocite (Cu_2_S) and covellite (CuS) are the two most common compounds of copper sulphide [49,120,121].

Copper sulphide formulas are typically written as Cu_2−x_S, where x has a value between 0 and 1 [121]. The composition stoichiometry of copper sulphide varies from Cu_2_S to CuS depending on the value of x [121]. Chalcocite copper sulphide (Cu_2_S) has different phases at different temperatures. Below 373 K, it is orthorhombic; between 373 and 700 K, it is hexagonal; and above 700 K, it is cubic [49,122]. Copper-rich sulphides, such as Cu_1.97_S, have a high *ZT* value of 1.7 at 1000 K [123]. The Crystal structure of different Cu_2−x_S components is shown in Figure 7f [124,125].

The thermopower and electrical conductivity of copper sulphide could be improved by phase tuning [49]. For example, phase transition effects the Seebeck coefficient of Cu_2−x_S (0 < x < 2), where it was found that the Seebeck coefficient of Cu_2_S is 270 μVK^−1^, whereas the Seebeck coefficient of the Cu_1.8_S is 20 μVK^−1^ [126]. Moreover, the doping approach could be a highly effective for improving TE performance. For example, *ZT* of 1.1 at 773 K has been produced from Na-doped Cu_9_S_5_ [127]. Nanostructuring could play a big role in enhancing the TE performance of copper sulphide [49,128]. For example, CuFeS_2_ nanocrystals with a diameter of 6.4 nm display a maximum *ZT* value of 0.264 at 500 K due to quantum confinement, which is 77 times the value of the bulk of CuFeS_2_ [128].

The electronic properties of copper sulphide TE materials could be enhanced by substituting one or more elements in their alloys. For example, dual replacement of In for Cu and Se for S increases thermopower and decreases lattice thermal conductivity, reaching *ZT* max = 1.0 in Cu_11.95_In_0.05_Sb_4_S_12.8_Se_0.2_, which is 56% higher than it is in Cu_12_Sb_4_S_13_ [129]. More information about the TE metal sulphides can be found in [49,130].

### 2.10. Half-Heusler Compounds

**Figure 8:**
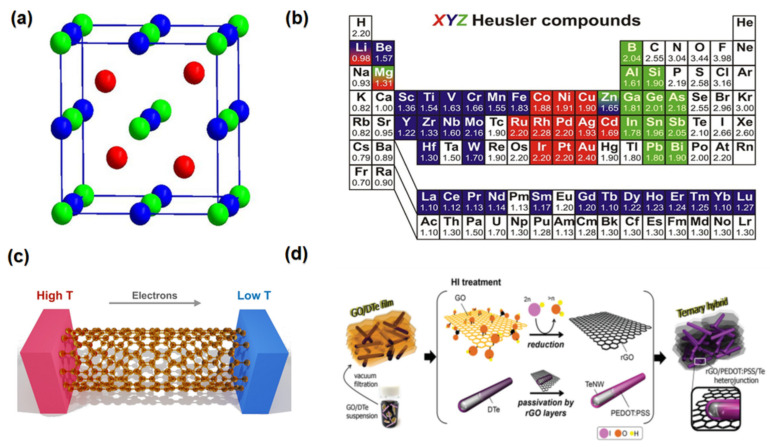
(**a**) Crystal structure of the Half-Heusler (MgAgAs type) phase. (**b**) The huge number of elements in the Periodic table of the elements that is possible to form Heusler compounds “Reproduced with permission from [164], Elsevier, 2019”. (**c**) TE carbon nano tube structure “Reproduced with permission from [144], MDPI, 2019”. (**d**) Schematic illustration of designed ternary TE composites paper “Reproduced with permission from [151], WILEY-VCH, 2016”.

Half-Heusler (HH) materials are desirable TE materials for medium-to-high-temperature applications [133]. This range of temperatures suits most industrial waste heat energy. In addition to their great TE performance, these materials have good mechanical strength, thermal stability, and low toxicity [133]. Half-Heuslers with the formula XYZ have a cubic MgAgAs type structure with four face-centred-cubic (fcc) sublattices, where three faces interpenetrate one, and there is one vacant sublattice; this is shown in Figure 8a, where X is the highest electronegative transition element, Y is the least electronegative transition element, and Z is a p-block element, such as Sn or Sb. Figure 8b illustrates the possible elements that can be used to form Heusler compounds [133,134].

Until recently, most of the research on Half-Heuslers was based on MCoSb, MNiSn, and NbFeSb, where M is Ti, Zr, or Hf. There are many options for tuning the electrical and lattice characteristics of Half-Heuslers, provided by the substitutability of the three lattice sites (X, Y, and Z). The most widely used n-type materials are compounds based on MNiSn, whereas MCoSb and XFeSb are often employed as p-type materials [55]. By using elemental substitution at the X and Y sites of Half-Heusler materials, mass fluctuation and strain field effects were used to lower the lattice thermal conductivity significantly [135]. For example, substituting Fe for the Co site in Zr_0.5_Hf_0.5_Fe_x_Co_1x_Sn_0.2_Sb_0.8_ reduces thermal conductivity to 3.35 Wm^−1^K^−1^ at 900 K under the arc melting process, which can promote *ZT* to 0.55 at 900 K [135]. Meanwhile, an elemental replacement was used at the X-site to adjust the carrier concentration and thus increase the electrical conductivity [136]. For example, *ZT* for Ti_0.25_Zr_0.75_NiSn_0.975_Ge_0.025_ at 775 K was significantly increased from 0.05 to 0.48 thanks to the pairing of a significant decrease in lattice thermal conductivity through mass fluctuation at the Ti/Zr site and an improvement of the thermopower and electrical conductivity through Ge substitution at the Sn site [136].

The *ZT* of Half-Heusler TE materials can be enhanced by nanocomposite by combining the arc-melting, ball-milling, and hot-pressing processes [55,133]. For instance, Giri Joshi et al. enhanced the *ZT* of Hf_0.75_Zr_0.25_NiSn_0.99_Sb_0.01_ from 0.8 to 1 using the nanocomposition approach [137]. Recently, the Half-Heusler TE family has been expanded due to the development of defective Half-Heusler compounds, including nominal 17- and 19-electron systems that have been shown to be good TE materials [138]. More information about the Half-Heusler TE can be found in [139].

### 2.11. Carbon-Based Materials

Over recent years, other promising state-of-the-art TE materials are carbon-based compounds, including CNTs, graphene, nanodiamonds, carbon fibre, and fullerenes [140]. Specifically, carbon nanotubes (CNTs), as shown in Figure 8c, and graphene have received more attention because of their high electrical conductivity and ability for nanostructure formation [141]. TE devices based on CNTs offer the potential for a wide range of applications, including low-temperature waste heat recovery [142].

Functionalised CNTs have electrical conductivities as high as 1500 Sm^−1^ and a Seebeck coefficient as high as 100 μVK^−1^ [143]. Moreover, carbon-based TE polymer composites are lightweight, inexpensive, flexible, and non-toxic, and this composition can be used to reduce the thermal conductivity of the carbon by coating its surfaces with the polymer matrix [33].

The 1D structure of CNTs makes them superior for the fabrication of TE materials, which exhibit better performance than bulk materials [144]. This performance can be increased by increasing the mobility or by reducing thermal conductivity via reducing their diameter. For example, composites of single-walled carbon nanotubes with organic/polymer compounds, such as poly (3,4-ethylenedioxythiophene): poly (styrene sulfonate), and/or polyvinyl acetate, demonstrated large TE power factors of ∼160 μW/m·K^2^; this is due to the weakly correlated of constant thermopowers with very high electrical conductivities, which makes the composites very promising for TE [145].

Recently, graphene has also been investigated extensively in TE due to its high electrical conductivity and potential TE properties when doped with atoms/molecules from other elements [146]. The few-layer graphene films with attached molecules enhanced the thermopower to more than 4.5 folds [147]. Another method to enhance thermopower is generating disorders in graphene, which is accomplished by treating graphene films with oxygen plasma resulting in an increase of the *ZT* up to three times [148]. Zhang wrote detailed information about organic TE materials [33].

### 2.12. Ternary Thermoelectric Composites

Polymers and organic materials have been used for electrical devices [149]. These materials fabricate wearable heating, cooling, and electrical generator TE devices that can operate at room temperature [42,49]. Their unique properties, such as having low thermal conductivity and being flexible, lightweight, non-toxic, and easy to fabricate, pique the interest of TE researchers [33]. However, they have a low Seebeck coefficient, power factor, and electrical conductivity [150]. Developing organic polymer-inorganic TE composite materials has overcome these limitations and provided excellent TE performance [150]. Therefore, ternary TE composites have become extensively researched in organic materials [33].

Ternary TE composites, such as the PEDOT: PSS/rGO/Te NWs, polypyrrole/graphene/PANi, and PEDOT: PSS/SWCNT/Te, have received a significant amount of attention due to their particular internal structure and the synergistic effect of its components [33]. For example, the power factor of a ternary hybrid composite of PEDOT: PSS/rGO/TeNWs, which is shown in Figure 8d, is 15 times higher than that of the binary film, which could be attributed to additional energy filtering at the interface between the rGO and PEDOT: PSS [151].

Printable TE composites made of PANi/graphene-PEDOT: PSS/PANi/double-walled CNT (DWNT) -PEDOT: PSS have been fabricated using a layer-by-layer assembly method. Then, by increasing the number of cycles, the electrical conductivity and Seebeck coefficient improved. The film’s high electrical conductivity and Seebeck coefficient gave it a TE power factor of 2710 μW/m·K^2^, higher than any other organic TE material measured at room temperature [152].

PEDOT/rGO/SWCNT ternary composites have been fabricated with high electrical conductivity compared to neat PEDOT and PEDOT/rGO binary composites because of the integrated electrical network, alignment of the PEDOT molecules, and change in molecular conformation. The electrical conductivity and Seebeck coefficient were further increased by treating PEDOT/rGO/SWCNT ternary composites with H_2_SO_4_. The power factor of the post-treated PEDOT/rGO/SWCNT ternary composite, which had 10% CNT, was 9.1 μW/m·K^2^, four times higher than the power factor without treatment [153].

### 2.13. Perovskite Materials

Perovskite is a material with a cubic crystal structure or non-cubic structure (orthorhombic, tetragonal phases). Perovskite follows the formula ABX_3_, where A is an organic or metal cation, B is a metal cation, and X is an anion (oxide or halide ion), which bonds to both cations [154]. It was discovered as perovskite, named after Russian mineralogist L. A. Perovski (1792–1856) [155]. The interest in perovskites has increased due to their wide range of unique properties, such as high conductivity, catalytic properties, and opto-electronic properties. They have been used in many applications, such as photovoltaics, light-emitting diodes, sensing, water splitting, etc. One of the interesting applications of perovskite is TE due to its TE characteristics and being cheap and quick to fabricate.

Oxide perovskite materials have been used as TE materials due to their high Seebeck coefficient. However, oxide perovskite materials have a low *ZT* due to their low electrical conductivity and high thermal conductivity. Single crystals of Sr(Mn_1−x_Mo_x_)O_3_, for example, have a *ZT*, Seebeck, electrical conductivity, and thermal conductivity values of 0.003, −120 μVK^−1^, 0.13 S.cm^−1^, and 5 Wm^−1^K^−1^, respectively, at 400 K [156]. It is possible to increase the electrical conductivity by doping. For example, in single-crystalline SrTiO_3_ doped with La, the electrical conductivity increased to 1000 S.cm^−1^, and *ZT* reached 0.08 at 750 k [111]. Electrical conductivity can be enhanced by reducing oxygen vacancy. When an oxygen vacancy is added to a perovskite material like ATiO_3_, two electrons are usually added to the Ti site. This creates charge carriers and increases electrical conductivity. For example, using pulsed laser deposition to introduce La and oxygen vacancies into SrTiO_3_, the electrical conductivity increased from 60 to 600 S.cm^−1^ at La 15% [157]. Oxygen vacancy also can be used to reduce thermal conductivity. For example, at room temperature, La-doped single-crystalline SrTiO_3_ decreased thermal conductivity by 40% due to oxygen vacancies acting as strong scattering centres for electrons and phonons [158].

In recent years, hybrid organic-inorganic perovskite materials have garnered considerable interest for TE applications because of their relatively high Seebeck coefficient of 2422 μVK^−1^ [159] and low thermal conductivity of 0.32 Wm^−1^K^−1^ at room temperature [160]. Theoretical findings show that hybrid perovskite materials could achieve a *ZT* value 0.95 [161]. However, they have low electrical conductivity compared with traditional TE materials. The hybrid perovskites TE materials’ electrical conductivity can be improved by doping [162]. More details about perovskite TE material can be found in [163].

Table 1 summarises the most well-known TE materials’ working temperature, performance, and properties. It can be noted that choosing suitable materials depends on the operation temperature, as *ZT* is temperature dependent. Moreover, to overcome the disadvantages of traditional materials, which are toxic, costly, and rare, it is necessary to choose eco-friendly, cheap materials and then develop their efficiency to compete with or overtake the efficiency of traditional TE materials.

## 3. Improvement Strategies for Thermoelectric Materials Performance

**Figure 9:**
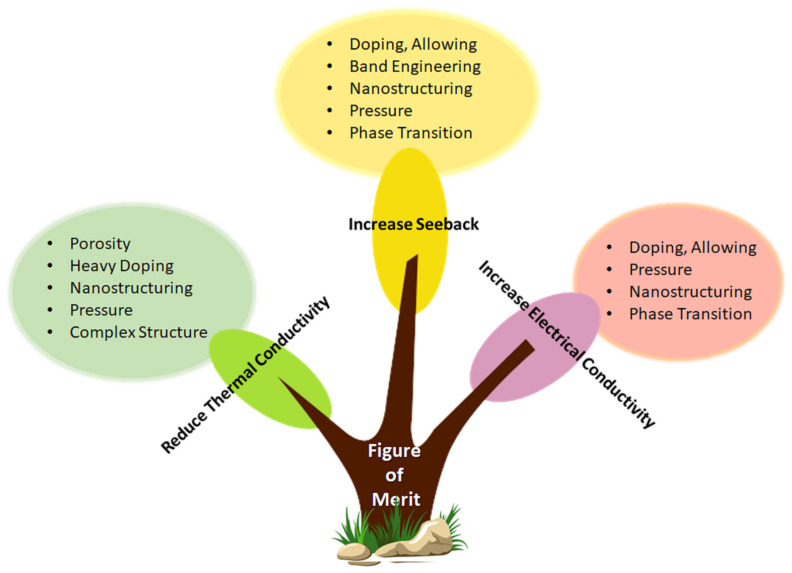
The most common methods for increasing the *ZT* value of thermoelements.

There are many modern concepts to optimise the transport parameters of TE material, such as doping, alloying, the fabrication of heavy element compounds, complex crystal structuring, resonant level doping, etc. One of the most effective methods is nanoscale approaches, such as nanostructuring, nanoinclusions, superlattices, NWs, and quantum dots, which are auspicious methods for increasing the performance of TE devices. Nanostructuring could be a remarkably effective tool to decouple the interdependence of TE parameters, which could highly increase the performance of TE materials. Figure 9 illustrates the different approaches to enhance the TE materials’ performance.

### 3.1. Alloying and Doping Approach

Introducing a point defect within the crystal lattice of materials results in a decrease in the average distance travelled by phonons, thereby reducing thermal conductivity. An effective approach to enhance the TE properties involves the incorporation of point defects through processes such as doping or alloying. This strategy aims to lower thermal conductivity while maintaining or even enhancing electrical conductivity.

The *ZT* of semiconductors is low because of their high thermal conductivity compared to their electrical conductivity. Alloying the material with the proper dopant can lower this ratio [181]. For example, the thermal conductivity of Ge can be reduced when it is doped with Si to make Si-Ge alloys. When Si is doped, a point defect is created that scatters the phonons, resulting in a huge decrease in thermal conductivity while increasing the overall TE efficiency. The reported thermal conductivity for pure Ge is around 55 Wm^−1^K^−1^, which decreases as the Si component increases. The lowest value measured for 40–60% Si in Ge is around 10–15 Wm^−1^K^−1^ [182]. As another example, doping Bi_2_Te_3_ with Cs will reduce the thermal conductivity of (CsBi_4_Te_6_) and increase the *ZT* to 0.8 below room temperature [183].

Adjusting the carrier concentration of the lattice thermal conduction will reduce the thermal conductivity without reducing the electrical conductivity. For example, adding Sb_2_Te_3_ and Bi_2_Se_3_ to Bi_2_Te_3_ reduces the lattice thermal conductivity without significantly affecting electronic properties, increasing the overall *ZT* [183]. Furthermore, doping creates extra energy levels between the bands, increasing electrical conductivity. For example, Peng et al. (2016) achieved a high *ZT* value of 1.2 at 300 K for p-type SnSe single crystals doped with Na, and *ZT* reached a peak of 2 at 800 K, compared to a ZT value of 0.23 for pure SnSe. This high value of the *ZT* is attributed to enhancing electrical conductivity without affecting the Seebeck [68]. Example is Duong et al. (2016) achieved *ZT* = 2.2 at 733 k with Bi-doped n-type SnSe single crystals due to enhanced electrical conductivity and maintained a low thermal conductivity of 0.46 Wm^−1^K^−1^ at room temperature [67].

The Seebeck coefficient could be increased by alloying and doping with an appropriate carrier concentration. The GeTe alloying and Ga doping of the n-type Pb_0.98_Ga_0.02_Te-5% GeTe achieved a high value of *ZT* of 1.47 at 673 K due to increasing the Seebeck coefficient by introducing the midgap states between a conduction band and valence band in PbTe, which in turn leads to an increased density of states (DOS) effective mass [184]. Moreover, the thermal conductivity drastically decreases to as low as 0.65 Wm^−1^K^−1^ at 62 due to enhanced phonon scattering taken on by the point defects and nanoscale Ga_2_Te_3_ precipitates [184].

Doping changes materials’ properties correspondingly, which may enlarge the power factor. For example, doping the In_0.53_Ga_0.47_ with ErAs nanoparticles improved the carrier concentration while maintaining high mobility, while the Seebeck coefficient was enhanced due to the effects of electron filtering [185]. For many components, it has been proven that the alloying effect applies not only to elemental mixtures and additional alloying of binary and ternary compounds. For example, the value of *ZT* of the CoAs_0.8_Sb_0.2_S alloy is increased almost 11% compared with pure CoAsS because the thermal conductivity is reduced by 44% [186].

### 3.2. Porosity Incorporation

**Figure 10:**
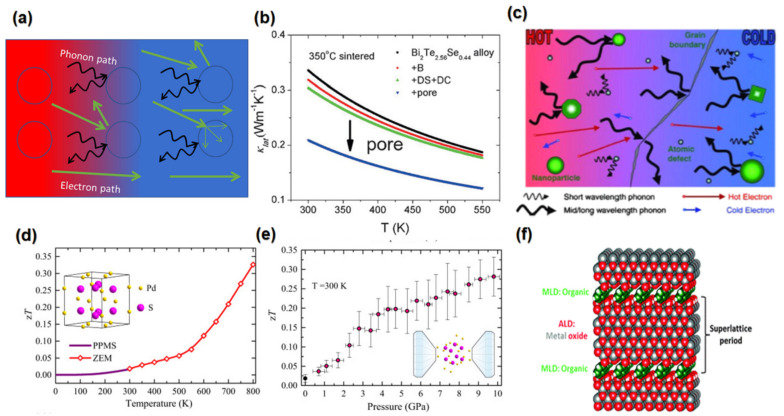
(**a**) The effect of the porosity on the phonon and electron path. (**b**) The effect of the porosity on the thermal conductivity Bi_2_Te_2.56_Se_0.44_ “Reproduced with permission from [187], WILEY-VCH, 2017”. (**c**) This simplified Figure depicts the mechanism of phonon scattering and the flow of hot and cold electrons through a TE material, “Reproduced with permission from [219], WILEY-VCH, 2010”. (**d**) Temperature dependence of *ZT* of PdS. (**e**) Pressure dependent *ZT* of PdS around room temperature “Reproduced with permission from [212], Elsevier, 2018”. (**f**) Structure of superlattice “Reproduced with permission from [215], Royal Society Of Chemistry, 2012”.

Porosity is commonly employed in structural designs to enhance the performance of TE materials by reducing thermal conductivity. Porosity is a very efficient tool for reducing thermal conductivity by phonon scattering, which occurs at the surface of the pores. Figure 10a shows the mechanism of phonon scattering by porosity. For example, Xu et al. (2017) made a theoretical calculation of the effect of the pores, grain boundaries, and dislocations on temperature-dependent thermal conductivity of Bi_2_Te_2.56_Se_0.44_ and found out pores extremely reduced the lattice thermal conductivity as shown in the Figure 10b [187].

Phonon scattering increase with the increase of crystal imperfection such as porosity, grain boundary, atomic defect, etc. However, the highest reduction of lattice thermal is by holes compared to other imperfections. Porosity is usually seen in polycrystalline materials during sintering processes. For example, introducing nanopores to SnSe can result in a high peak *ZT* of 1.7 at 823 K, as nanoporosity reduces thermal conductivity up to 0.24 Wm^–1^ K^–1^ [188]. There are many approaches to producing porosity; the most common is sintering. For example, highly porose Bi_2_Te_2.5_Se_0.5_ hollow nanorods achieve a low thermal conductivity of 0.13 Wm^−1^K^−1^, which leads to a high *ZT* of 1 at 488 k for sintering conditions of 400 °C, 40 MPa, 5 min [187]. Moreover, nanoporosity could be obtained by synthesis instead of sintering [189]. However, the porosity could increase the electrical resistivity because pores could block carrier transport, as seen clearly in Figure 10a [190]. Unexpectedly, Haihua et al. (2021) introduced pore networks into Cu_12_Sb_4_S_13_ by sublimating 0.7% BiI_3_ during annealing; the thermal conductivity reduced to 72% and, at the same time, the electrical conductivity increased due to an increase in the carrier mobility [191].

### 3.3. Heavy Element Addition

Most compounds with heavy elements have limited phonon propagation, which helps to lower the thermal conductivity of the lattice. This is obvious from the features of significant TE compounds containing heavy elements, such as Bi_2_Te_3_, PbTe, BiSb, etc. Heavy components are the primary reason these have low heat transfer rates despite their high electrical conductivity.

Semiconductor doping with heavy elements (with carrier concentrations ranging from 1019 to 1021 per cm^3^) has also shown potential for increasing the TE properties of many materials. For example, it is possible to enhance the TE properties of ZnO by doping with In, which will reduce thermal conductivity from (60 Wm^−1^ K^−1^) to (3 Wm^−1^ K^−1^), leading to a good *ZT* of 0.45 at 1000 K [192].

Generally, heavier atoms are assumed to reduce lattice thermal conductivity more effectively. For example, the lattice thermal conductivity is reduced dramatically to a value of 1.8 Wm^−1^ K^−1^ at room temperature by Eu-filling for Eu_0.34_Co_4_Sb_12_ [193]. Moreover, when heavy metals like Pb are doped into BiCuSeO ceramics, the electrical conductivity will increase, and the thermal conductivity will be reduced, leading to a high *ZT* value of 1.1 at 823 K [194].

### 3.4. Band Engineering Approaches

Band engineering approaches in TE materials, such as resonant level doping, electronic band convergence, and band curvedness, have improved the power factor. When the energy level of the dopant is close to the valence band or conduction band, this circumstance may result in adding extra energy levels to the host band, resulting in a distortion of the electronic DOS that is known as resonant doping [195,196,197]. This distortion may modify the DOS when the Fermi level is close to the resonant state, which increases the density of states without changing the carrier concentration significantly, resulting in a significant increase in the Seebeck coefficient, as demonstrated in Equation (6) [198].

Widespread doping of Ga, In, and Ti in the classical semiconductor TE material (PbTe) will produce resonance energy levels. The high *ZT* value reported in Ti-doped PbTe of 1.5 compared with 0.7 on undoped PbTe is an example of this and is caused by resonant levels produced by the Ti [199]. SnTe_0⋅85_Se_0.15_ doped with 1.5 mol% In increased the Seebeck coefficient to 175 μVK^−1^ and *ZT* to 0.8 at 855 K. This is a result of the resonance level caused by In dopants [200].

However, the DOS modification may reduce mobility as mobility is inversely proportional to the band-mass (m_b_*) of a single valley, as described in Equation (13) [201]

(13) $$\mu \;\propto \frac{1}{m_{b}{}^{*5/2}}$$

Therefore, low m_b_* is necessary to improve the TE performance of materials.

The relation between m_b_*and the DOSs effective mass (m*) can be expressed by the following equation, as described in Equation (14) [201]

(14) $$m^{*}=N_{v}{}^{2/3}m_{b}{}^{*}$$

whereas $N_{v\;}$ is valley degeneracy (orbital).

Furthermore, *m*_b_*and m* have opposing effects on the enhancement of *ZT* since large mb* reduces carrier mobility, which in turn decreases electrical conductivity; contrarily, large m* enhances the power factor [202]. For example, when PbTe is doped with La and I, it is found that La-doped PbTe increases m* more than I-doped PbTe increases m*, resulting in the greater *ZT* seen in I-doped PbTe of 1.4 compared to La-doped PbTe at 800 K [203].

Carrier pocket engineering can be used to increase m* without decreasing μ when symmetrically different bands (high N_v_) in low-dimensional TE materials come together. The effective convergence of the bands results in an effective increase in *Nv*. For example, In PbTe_1−x_Se_x_ alloys has two valence bands at L where the valley degeneracy of 4 and Σ is 12. To increase the TE performance, the convergence of many valleys has been created at high temperatures by Se alloying, resulting in increased valley degeneracy Nv (16 in total) at high temperatures; this will lead to an ultrahigh *ZT* of 1.8 at 850 K [204].

The effective mass is proportional to the band’s curvature (effective mass ∝ 1/curvature). When the energy band distortionenergy band distortion reshapes the bandgap reshapes the bandgap, it results in a rise in the DOS at the Fermi level [199]. Moreover, band flattening is frequently used to improve the effective mass. Band flattening can be induced by providing dopants that accommodate extremely concentrated orbitals [205]. Thus, when La or Nd substitutes Sr in SrTiO_3_ perovskites, the number of overlapping orbitals decreases. Consequently, in La-doped PbTe, convergence between La f states and Pb p states can influence the conduction band at the L point [203].

### 3.5. Phonon-Glass Electron-Crystal (PGEC)

Heat is conveyed mostly by diverse phonons with different mean free pathways, as shown in Figure 10c. Slack suggested that Phonon-Glass Electron-Crystal (PGEC) materials can reduce lattice thermal conductivity by scattering phonons without impacting electron transport [23]. Because of the lack of an ordered crystal structure in glasses, they have the lowest thermal conductivity, whereas crystalline materials promote electrical conductivity [206]. Slack suggests a method to design a complex crystal structure using TE materials. This method has proven to be effective; due to the complexity of their crystal structures, these materials have low thermal and high electrical conductivity, which results from their highly periodic crystal ordering [23,31].

Clathrates, skutterudites, and Zintl phases have been studied intensively and modified by doping and alloying to work according to the PGEC concept [181,207]. For example, because of the complex structure of CsBi_4_Te _8_, it has a lower lattice thermal conductivity of 1.1 Wm^−1^K^−1^ than Bi_2_Te_3_, and thus an enhanced *ZT* of 0.8 below room temperature [31]. The complex Zintl phase of p-type Yb_14_MnSb_11_ permits an extremely low thermal conductivity of 0.4 Wm^−1^K^−1^, which leads to *ZT*=1 at 1223 K [92]. Also, hybrid TE materials of amorphous/crystalline composite structures possess high PGEC. For example, Ag_4.02_TeS amorphous/crystalline composites have deficient thermal conductivity value of 0.07 Wm^−1^K^−1^ and achieve a *ZT* value of 0.58 at room temperature [208].

### 3.6. Pressure-Induced Approach

High-pressure technology provides an interesting potential for developing high-performance TE materials. Material under hydrostatic pressure can be a strong tool for improving TE performance, By altering the crystal structure and electronic structure without introducing impurities, causing simultaneous changes to their chemical and physical characteristics [209]. For example, a significant improvement in the TE characteristics of p-doped Sb_1.5_Bi_0.5_Te_3_. The Seebeck coefficient increases from 212 to 305 μVK^−1^ at 1.7 GPa, resulting in an increased *ZT* value of 2 [210].

Synthesis of material in the thin stressed film will obtain similar effects because there will be improvements in the texture and preferred orientation, leading to enhanced electrical conductivity [209]. For example, Bi_2_Te_3_ have its maximum *ZT* values when synthesised at 2.5 GPa [211]. Furthermore, the pressure-induced enhancement led to an increase in the power factor and a decrease in the thermal conductivity of PdS. As a result, the *ZT* value at room temperature under 10 Gpa pressure was the same as the *ZT* value at a high temperature of 800 K, as illustrated in Figure 10d,e [212].

### 3.7. Superlattice Approach

Superlattice is the periodic layering of two or more substances with a several nanometre thicknesses, as shown in Figure 10f [42]. Superlattices could enhance the *ZT* value by decreasing the phonon thermal conductivity [213]. A total of 90% of the thermal conductivity in semiconductors is typically provided by lattice thermal conductivity, so lowering lattice thermal conductivity will improve TE performance [214]. The superlattices can significantly reduce lattice thermal conductivity compared to the bulk material because of the scattering phonons at interfaces between layers [215]. Moreover, there will be a high reduction of anisotropy in lattice thermal conductivity as the phonon scattering will be at a maximum in that direction perpendicular to the superlattice interfaces, which is favourable for TE applications.

The alternating layers of various materials may inhibit phonon transmission by internal interface scattering, and the electronic characteristics can stay essentially interactive if the superlattice’s periodicity is suitably tuned [40]. A study of phonon transmission throughout a sandwich of superlattice layers shows that increasing the number of layers results in a greater phonon scattering effect because of the interruption of phonon propagation at each interface, resulting in extremely low lattice thermal conductivity [216]. For example, in Bi_2_Te_3/_Sb_2_Te_3_ superlattices, *ZT* can be enhanced by up to 2–3 times [217].

Due to the quantum confinement effect, the number of states near the Fermi level increases, which gives the superlatticthe potential to enhance the Seebeck coefficient [218]. The TE performance of superlattice thin films has been demonstrated to be outstanding. For example, PbTeSe/PbTe superlattice thin films show ultrahigh *ZT* values of >2 [190].

### 3.8. Phase Transition

A phase transition refers to the phenomenon in which a material undergoes a change in its state from one phase characterised by specific properties to another phase with distinct properties. In TE materials, a broad temperature range is typically involved, and certain materials exhibit different phases at different temperatures, leading to diverse TE performance. To achieve optimal TE performance within the desired temperature range, it is crucial to deliberately design and induce a phase transition that yields a phase possessing superior TE properties.

Annealing could be used to achieve the proper phase with high TE performance; for example, Siyar et al. (2018) investigated the effect of annealing temperature on the phase transition of Cu_2_SnSe_3_ and found that the Cu_2_SnSe_3_ annealed at 960 K was mostly cubic, while at 600 K, it was a monoclinic phase. The *ZT* of cubic Cu_2_SnSe_3_ has a higher *ZT* (0.09) than the monoclinic phase. Cubic Cu_2_SnSe_3_ has a narrower band gap (0.92 eV) than monoclinic Cu_2_SnSe_3_ (1.00 eV) at ambient temperature, which contributes to the superior TE performance of the cubic phase [220].

Synthesis methods can produce different primary and secondary phases to reach the required phase by choosing the proper synthesis method. For example, Thakur et al. (2020) used different routes and profiles to make (Bi_2_Te_3_) nanoparticles to achieve the best pure single phase. They demonstrated that only a small amount (1.6%) of Bi_2_Te_3_ was formed in the coprecipitation method. At the same time, the hydrothermal technique increased this phase with an increment in synthesis duration, which resulted in an increase in the electrical conductivity by 20 times, and this result contributed to the secondary phases that were produced, such as BiTe, Bi_4_Te_3_, and TeO_2_, which caused the lowering of the band gap [221].

**Figure 11:**
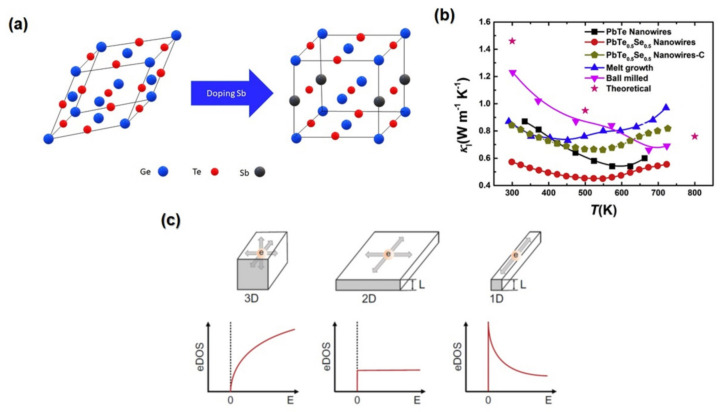
(**a**) The phase transition between the rhombohedral and cubic phase in Sb-doped GeTe. (**b**) The schematic diagram illustrates the thermal conductivity of PbTe NWs and PbTe_0.5_Se_0.5_ bulks “Reproduced with permission from [224], Elsevier, 2020”. (**c**) The density of states (DOS) of bulk (3D), 2D, and 1D materials as a function of energy and, by lowering the dimensions, the (DOS) is enhanced. “Reproduced with permission from [234], WILEY-VCH, 2020”.

TE material properties can be improved by tuning the phase transition temperature, which is possible by doing. For example, Jin et al. (2021) tuned the phase transition temperature to enhance the electrical and thermal transport properties of GeTe by doping with Sb. They found that the phase transition temperature decreases from ∼673 K to ∼580 K by entropy augmenting through doping with Sb, which enlarges the crystal symmetry of GeTe, resulting in an enhancement of the band degeneracy as the phase transition from the rhombohedral and cubic phase in Sb-doped GeTe, as shown in Figure 11a. Sb counter-doping significantly raises the Seebeck coefficient by adjusting the carrier concentration and flattening the valence band, resulting in a maximum *ZT* of 1.8 at 773 K, which is achieved in Ge_0.9_Sb_0.1_Te [222].

### 3.9. Nanostructuring Approach

One of the most important and desirable forms of materials is nanomaterials. These materials have structures where, at minimum, the scale of one of their dimensions ranges between 1 and 1000 nm. Hicks and Dresselhaus demonstrated by theoretical calculations that the TE properties of low-dimensional nanostructured materials are much superior to those of the same bulk materials [28]. Nanostructuring is an effective approach for reducing the thermal conductivity of lattices without reducing the power factor because this method enhances phonon scattering by increasing the interfaces [223]. For example, the ultrathin PbTe_0_._5_Se_0.5_ NWs synthesised by solution had a very low thermal conductivity of 0.45 Wm^−1^K^−1^ [224]. Figure 11b shows the reduction of thermal conductivity by 1D nanostructured PbTe relative to their bulk equivalents.

As system dimensions reach the nanometre scale in these materials, its TE performance notably improves because of the quantum size effect [225]. The quantum size effect means that the electronic and the physical properties of the material are alerted when the size reaches the nanoscale because the nanostructures restrict the degree of freedom of the carrier’s motion in certain paths, which then leads to considerable alterations in the electronic transport’s behaviour [226]. It can be seen clearly in Figure 11c that there are dramatic changes in the electronic density of states. The density of states in 1D materials is very sharp compared with 3D materials. That will offer the possibility for enhanced TE parameters, especially the Seebeck coefficient [227].

Through quantum confinement, nanostructuring can enhance the DOS around the Fermi level, improving the Seebeck and affording a mechanism to decouple the thermal and electrical conductivity [228,229]. For example, a high power factor of ∼25 μW cm^−1^ K^−2^ in the in-plane direction at room temperature has been achieved for Si-rich SiGe/Si superlattices with controlled interfaces, which also exhibited a low thermal conductivity of 2.5 Wm^−1^K^−1^ [230].

In semiconductors with high doping levels, the average distance electrons travel is considerably shorter than that of photons. However, by fabricating nanostructures with one or more dimensions, it becomes feasible to create many surfaces where phonons (vibrational energy quanta) can be scattered more effectively and selectively compared to electrons, even across a wide range of distances. As a result, the lattice thermal conductivity can be significantly reduced while maintaining high carrier mobility and efficient electronic conduction [228]. This can be done by creating nanostructures with one or more dimensions less than the mean free path of phonons but bigger than that of charge carriers [231].

Enhancing a material’s TE properties by adjusting the lattice inclusions’ size is possible. Because thermal conductivity will be reduced due to plenty of interfaces being formed, and the power factor will also be improved due to energy filtering and electron injection phenomena [232], such as, a high *ZT* of 1.5 at 750 K achieved due to lower lattice thermal conductivity by hierarchical phonon scattering and an increased Seebeck coefficient by energy filtering for Te nanoinclusions that is generated in-situ in SnTe [233]. Based on the preceding analysis, it can be deduced that the nanostructuring process can significantly decrease thermal conductivity, amplify the Seebeck coefficient, and uphold or potentially augment the electrical conductivity of TE materials. These outcomes collectively indicate that nanostructuring emerges as a highly effective approach in mitigating the interdependencies among TE parameters, rendering it one of the most promising methodologies in the field.

## 4. One-Dimensional Thermoelectric Materials

The field of TE conversion technology is confronted with three primary limitations. Firstly, the conversion efficiency of TE devices is often low, posing a significant hurdle in their application for electrical power generation. Secondly, TE materials with high efficiency are rare, expensive, and often toxic, further hindering their widespread utilization. Lastly, the design and manufacturing of TE devices are complex and costly.

It is crucial to explore alternative approaches to address these challenges and enable the large-scale implementation of TE devices. One potential solution lies in selecting inexpensive, abundant, and environmentally friendly materials, coupled with efforts to enhance their efficiency. Additionally, the development of fabrication methods suitable for large-scale production is imperative.

To this end, reducing the dimensions of TE materials offers a promising avenue for improving their efficiency across a wide range of material types. In particular, 1D nanostructured TE materials have emerged as highly interesting and influential nanostructures in this regard. By leveraging these advancements, it becomes feasible to overcome the limitations associated with TE conversion technology and unlock the potential for large-scale TE power generation.

Reduced material dimensions will change the state density of electrons, which will modify energy bands and lead to variable carrier mobility. Since the size of 1D nanostructures is smaller than the mean free path of phonons, boundary scattering is amplified, and thermal conductivity could be decreased [85]. At the same time, the 1D carrier transport could be maintained or enhanced, keeping the high electrical conductivity of the original material or improving it significantly. Moreover, the Seebeck coefficient of a material might potentially be increased by quantum confinement effects [85]. As a result, 1D TE material could have larger *ZT* than bulk material.

This section provides a comprehensive discussion of the 1D configurations of TE materials, highlighting the influence of 1D nanostructures on the TE performance. Furthermore, it elucidates the most important large-scale synthesis techniques for producing 1D nanostructure TE materials, along with an exploration of the methodologies employed for measuring the performance of TE materials with 1D nanostructures.

### 4.1. Configuration of One-Dimensional Thermoelectric Materials

#### 4.1.1. Quantum Wires

Quantum wires (QWs) are semiconductor particles that measure less than 10 nanometres in a single dimension. Due to their different bandgap, QWs exhibit strong quantum effects that influence transport properties [42]. According to theoretical research, greater electron confinement will significantly improve *ZT* inside quantum wires [28]. Some material systems with small electron-effective masses, such as InSb, have the potential to achieve high power factor and *ZT* values with diameters of (>5 nm) [235,236].

**Figure 12:**
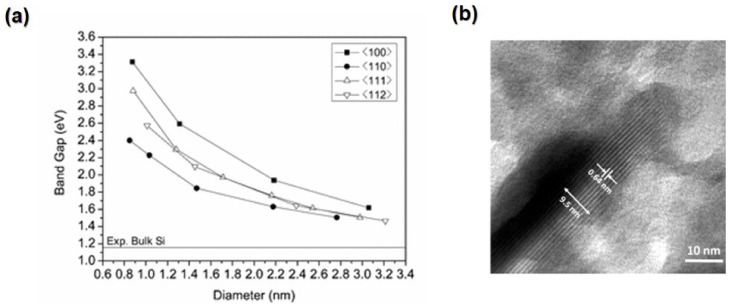
(**a**) effect the quantum wire diameter of Si on the band gap “Reproduced with permission from [237] American Physical Society, 2007” (**b**) TEM image of 9.5 nm diameter QWs “Reproduced with permission from [244], American Chemical Society, 2021”.

The theoretical *ZT* value of Bi_2_Te_3_ QWs with a diameter of 0.5 nm is more than 14, demonstrating that QWs have a very high potential for use in TE devices and need more attention [28]. Furthermore, QWs have distinct electrical properties that distinguish them from larger particles, making them a candidate for use in TE devices, mainly for two dimensions and flexible TEGs. Figure 12a illustrates the computed bandgap as a proportion of diameter for Si QWs in various orientations. It can be seen clearly that the bandgap of Si QWs fluctuates with the orientation, revealing a direction-dependent behaviour. Due to the quantum confinement effect, the bandgap values dropped with increasing QW diameter [237]. The calculated current with a voltage of Si QWs shows that the current increase, which occurs when the diameter of the Si QWs is reduced, indicates that QWs can significantly enhance the electrical transport performance [238]. Figure 12b shows a TEM image of 9.5 nm diameter single-crystallinity QWs.

QWs can be synthesised using a variety of synthetic techniques. For example, Si QW could be fabricated using the vapour-liquid-solid (VLS) growth method [238]. CsPbBr_3_ QWs with a diameter range from 10 to 3.4 nm from the nonconfined regime to the strong quantum-confinement regime, respectively, have been produced using a colloidal synthesis technique where the diameter of the CsPbBr_3_ QWs can be controlled by adjusting the short acid and the amine ligand ratio, the reaction time, and the temperature [239]. Theoretically, when the diameter of ZnO QWs was 0.8 nm, *ZT* rose 30 times compared to bulk [240]. Li et al. (2019) fabricated single-unit-cell ZnS QWs less than 1 nm in diameter by synergizing a soft template with oriented attachment (STOA) [241]. Moreover, Zn with 4 nm QWs nanocomposites synthesised by thermal evaporation with porous host materials shows unique TE properties with a very high room temperature Seebeck coefficient of 130 μVK^−1^ [242].

The *ZT* of Si/Ge super lattice quantum wires with a 1.7 nm width > 1 at 300 K can be attributed to the 3-fold reduction of thermal conductivity compared to bulk Si [243], while the maximum *ZT* of SiGe quantum wires may be greater than 2, according to theoretical calculations [80].

#### 4.1.2. Nanowires and Nanorods

**Figure 13:**
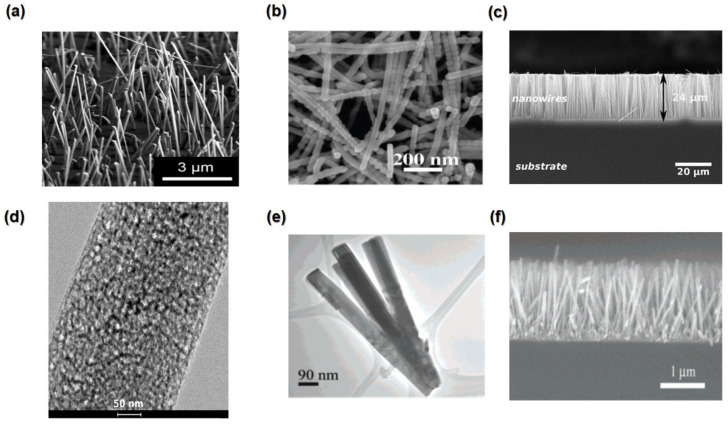
(**a**) SEM image of Bi nanowires “Reproduced with permission from [262], American Chemical Society, 2014”. (**b**) The pearl-necklace-shaped PbTe NWs “Reproduced with permission from [255], American Chemical Society, 2008”. (**c**) Si NWs array. (**d**) Si nanowire and its roughness are clearly seen at the surface of the wire “Reproduced with permission from [257], American Chemical Society, 2020”. (**e**) ZnO NWs (**f**) ZnO NWs array “Reproduced with permission from [263], American Chemical Society, 2006”.

NWs and NRs are 1D nanocrystals, like QWs, but are substantially higher in diameter. According to theoretical calculations, n-type Bi NWs can reach a *ZT* of around 6 at 77 K [245]. Seebeck coefficients for Bi NWs with diameters of less than 50 nm are expected to be significantly higher than those in bulk, mainly attributed to the quantum confinement effect. Single crystalline Bi NWs have been shown to have a thermal conductivity of 3–6 times less than bulk between 100 to 300 K [246]. Polycrystalline Bi NWs with a 74–255 nm diameter demonstrate 18–78 times lower thermal conductivities than bulk Bi NWs throughout the same temperature range [246]. Single crystalline Bi NWs with 40 nm diameter have extremely low lattice thermal conductivity of 0.13 Wm^−1^K^−1^ at 77 K [247], while the Bi_2_Sb_0.6_Te_3_ NWs showed a high Seebeck coefficient of −630 μVK^−1^ at 300 K [248]. Figure 13a shows an SEM image of the synthesis of Bi nanowire arrays by physical vapour deposition. Jigui et al. (2023) fabricated flexible TEGs from bismuth telluride nanorods. The flexible devices were each made of five generators; each wasmade of n-type material (Bi_2_Te_2.7_Se_0.3_) and p-type (Bi_0.5_Sb_1.5_Te_3_)_._ These flexible TEGs gave a maximum power of 6.06 Wm^−2^ at temperature differences of 28.3 K [249].

NWS and NRs could be very effective tools to increase the efficiency of various TE materials. For example, GaAs NWs grown by gallium-assisted molecular beam epitaxy exhibit very high structural quality, with diameters between 150 and 170 nm and TE conductivity of 8–36 Wm^−1^K^−1^ [250]. The SiGe alloy NWs grown by the VLS method achieved a large TE *ZT* of 0.46 compared with bulk SiGe, resulting from the low thermal conductivity of 1.2 W/m^−1^K^−1^ at 450 K [80]. β-SiC NWs grown by CVD resulted in 60–100 nm diameter with a low thermal conductivity of 86.5 Wm^−1^K^−1^ and a Seebeck of −1.21 μVK^−1^, resulting in a *ZT* value of 0.12, which is 120 times that of bulk β-SiC [251]. Y Wang and HJ Fan synthesised the single-crystalline La_1−x_Sr_x_CoO_3_ nanowire by the hydrothermal method and obtained a *ZT* of 0.19 at 300 K, twice that in bulk [252]. Bui et al. synthesised oxide (ZnO) NWs (NWs) with diameters ranging from 50 to 210 nm using CVD and investigated the diameter-dependent thermal transport, concluding that there is a significant reduction in thermal conductivity of (ZnO) NWs (NWs) compared to bulk ZnO due to increased phonon boundary scattering [253]. Rusiri et al. (2022) investigated the effect of ZnO morphology, such as nanoribbons, nanorods, nanoparticles, and nanoshuttles, on the TE performance and found that the highest performance can be achieved using ZnO NRDs [254].

The roughness of the NWs could enhance the TE material’s performance. For example, using a hydrothermal technique effectively synthesised uniform single-crystal pearl-necklace-shaped PbTe NWs, as shown in Figure 13b, with an average diameter of around 30 nm and a Seebeck coefficient of around 307 μVK^−1^, which is approximately 16% more than that of bulk PbTe at ambient temperature [255]. The rough silicon NWs’ electrochemical synthesis has low thermal conductivity and a good *ZT* of 0.6 at room temperature [256]. Figure 13c,d shows a rough surface silicon NW with relatively low thermal conductivity [257].

Doping NWs with the proper element could boost the efficiency of the TE materials. For example, the Seebeck and electrical conductivity of n-doped InSb NWs with (43 nm) diameters grown using the VLS method show lower Seebeck values but larger electrical conductivity values than those of pure bulk crystals [258]. Se-doped InSb NWs grown by Au-assisted chemical beam epitaxy have diameters ranging from 80 nm to 200 nm and achieve a low of *ZT* = 0.025 [259]. Solution-phase synthesis was used to grow InAs NWs 20 nm in diameter, demonstrating a significant improvement in TE properties due to the quantum confinement effect [260]. Polytypoid M_2_O_3_(ZnO) NWs, where M (In, Ga, Fe) was synthesised using the VLS method, demonstrated reduced thermal conductivity. Figure 13e,f shows NWs array and multitube NWs of ZnO. Due to the polytypoid structure, *ZT* was increased from 1.7 × 10^−4^ for ZnO NWs to 0.055 for the IGZO NWs at 300 k due to the phonon scattering at composite interfaces [261].

#### 4.1.3. Nanofibers

Nanofibers, characterised by a diameter of less than 1000 nm, have attracted significant attention due to their remarkable flexibility, lightweight nature, and versatile fabrication capabilities in textiles. They have found extensive applications in tissue engineering technology, particularly for developing portable and wearable TE materials. Nanofibers can be fabricated through various methods, including electrospinning, a two-step anodic oxidation process, dissolved phase deposition, and thermal drawing. Depending on their composition, nanofibers used in TE materials can be classified into three categories: organic fibres, inorganic fibres, and inorganic/organic hybrid fibres.

**Figure 14:**
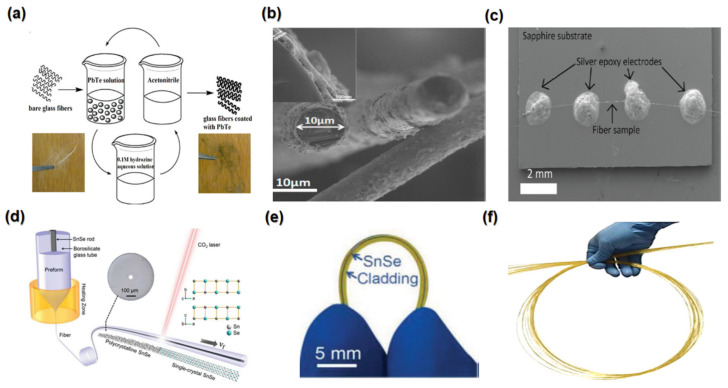
(**a**) The procedure of coating glass fibre with PbTe. (**b**) PbTe nanocrystal-coated glass fibres “Reproduced with permission from [266], American Chemical Society, 2012” (**c**) SEM image of the fibre connected by electrodes on substrate for thermal conductivity measurements, “Reproduced with permission from [267], American Chemical Society, 2013”. (**d**) Schematic of thermal drawing of SnSe fibre and post-draw laser recrystallization process (**e**) Single SnSe fibre with good flexibility (**f**) Photograph of SnSe fibres that are tens of metres long “Reproduced with permission from [270], WILEY-VCH, 2020”.

1D TE fibre fabricated by the deposition of nickel and silver as strips on silicon fibre can achieve a Seebeck coefficient of 19.6 µV K^–1^ [264]. Woven-yarn TE textiles were fabricated by the deposition of Sb_2_Te_3_ (p-type) and Bi_2_Te_3_ (n-type) on PAN nanofibers, and their figures of merit were 0.24 and 0.07, respectively [265]. Liang et al. (2012) deposited thick PbTe nanocrystals on glass fibres, as shown in Figure 14a,b, which enhanced the TE properties and flexibility and achieved a *ZT* of 0.73 at 400 K [266]. Figure 14c shows an SEM Image of the device setup to characterise the TE properties of PbTe nanofiber [267].

A novel glass-fibre-aided cold-press method was developed to achieve flexible n-type Ag_2_Te nanowire (NW) films [268]. These films exhibit a significant increase in electrical conductivity and the highest power factor, value up to 192 μW/m·K^2^ at 470 K, due to the disappearance of grain boundaries under compression to 30 MPa [268]. A special drawing technique of glass-cladding fibres was used to fabricate polycrystalline continuous Bi_2_Te_3_-core fibre. The Bi_2_Te_3_ core enhanced the electrical conductivity and achieved a high *ZT* of 0.73 at 300 K [269].

Single-crystal SnSe TE fibre was fabricated using laser-induced synthesis. SnSe was thermally drawn into a highly flexible, ultralong, and mechanically stable polycrystalline fibre, then treated with a CO_2_ laser to recrystallise the SnSe core to single-crystal over the entire fibre, as shown in Figure 14d–f. The ultralong single-crystal rock-salt SnSe fibres possess high TE properties, significantly enhancing the *ZT* value up to 2 at 862 K. Then, a shirt fabricated from the TE SnSe fibres produced 30 mV due to the temperature difference between the skin and the environment [270].

Ca_3_Co_4_O_9_ nanofibers with tiny grain sizes were synthesised using a sol-gel-based electrospinning technique and then consolidated into bulk ceramics using spark plasma, resulting in a 55% increase in *ZT* of 0.40 at 975 K [271]. La_0.95_Sr_0.05_CoO_3_ nanofibers with a diameter of ∼35 nm were synthesised by electrospinning and obtained a Seebeck coefficient of 650 μV K^−1^ at room temperature [272].

#### 4.1.4. Heterostructures

Heterostructures composed of materials with varying dimensionalities, such as 0D, 1D, and 2D, have gotten significant attention because of their great potential for various applications [273]. Heterostructure TE materials, such as multisegmented and core/shell NWs, have piqued considerable attention due to the possibility of more efficient tuning of the TE performance. Compared to NWs, the heterostructure interface between the core and shell and between every two adjacent segments in a multisegmented NW can further decrease the lattice thermal conductivity by preventing phonon scattering down the wire axis without affecting electrical conduction [85].

**Figure 15:**
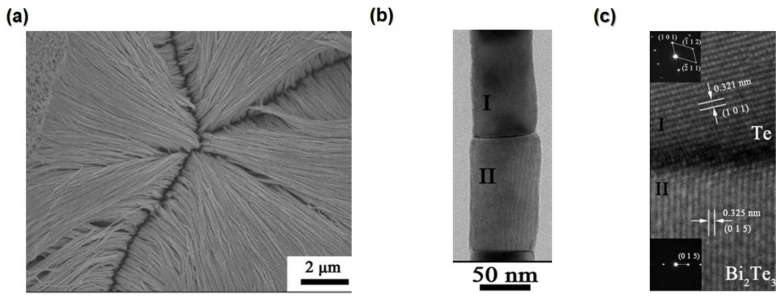
(**a**) The Bi_2_Te_3_/Te NWs array (**b**) Close up on Bi_2_Te_3_(II)/Te(I) segments (**c**) HRTEM image and SAED patterns of each segment “Reproduced with permission from [274], American Chemical Society, 2007”.

Many chemical processes, such as electrodeposition, solution-phase epitaxial growth, etc., can synthesise heterostructure NWs. Wang et al. (2008) synthesised single-crystal Bi_2_Te_3_ and Te segments, as shown in Figure 15a–c, by using a thermal annealing process where precipitation is confined using porous anodic alumina (PAA) membranes [274]. Moreover, Li et al. (2011) fabricated Bi_2_Te_2_Se/Te segments of NWs with diameters of 60–85 nm and lengths of 20 μm using the AAO template-assisted pulsed electrodeposition technique, and found that the length and structure of the segments can be modified by adjusting the precursor concentration [275].

A theoretical study shows the effect of germanium (Ge) coatings on the thermal transport properties of silicon (Si) NWs. This coating causes a dramatic decrease in thermal conductivity, down to 75% at room temperature, compared to uncoated Si NWs [276]. Another study calculated the TE *ZT* for p-type Si NWs with axial Ge heterostructures and found the *ZT* could reach a high value of 3 in the direction of ⟨111⟩ [277]. However, the synthesis of core/shell NWs is still significantly more challenging than axial 1D heterostructures. Core/shell NWs can be synthesised by on-film techniques [278], chemical vapour deposition [279], and the solution-phase method [280]. Te/Bi core/shell NWs with small diameters (20–25 nm) synthesised by the solution phase epitaxial process have a much higher Seebeck coefficient and a lower thermal conductivity of –128 μVK^−1^ and 0.55 Wm^−1^K^−1^, respectively, at 300 K [280].

#### 4.1.5. Nanotubes

**Figure 16:**
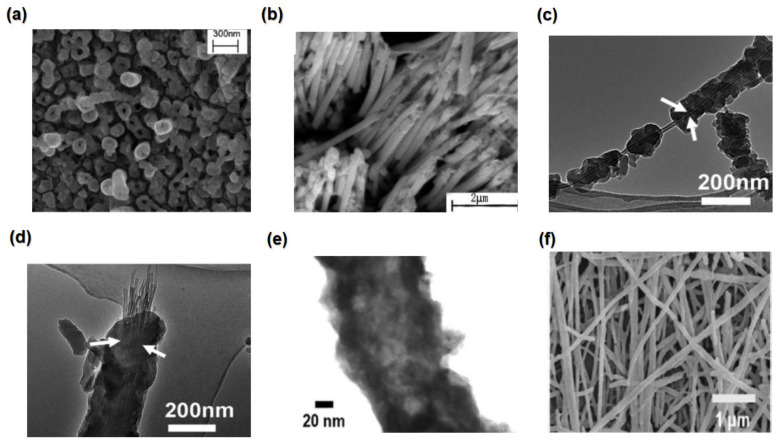
(**a**) (Bi_2_Te_3-y_Se_y_) nanotubes. (**b**) (Bi_2−x_Sb_x_Te_3_) nanotubes “Reproduced with permission from [284], American Chemical Society, 2008”. (**c**) TEM images of individual CNTs coated with a PANI layer that indicates by white arrows. (**d**) CNT bundles coated with a PANI layer that indicates by white arrows “Reproduced with permission from [290], WILEY-VCH, 2010”. (**e**) PbTe nanotubes. (**f**) PbTe single nanotube “Reproduced with permission from [292], Elsevier, 2018”.

Nanotube TE materials have attracted great interest as promising high-efficiency TE materials due to strong phonon scattering and their low-dimension, hollow tubular morphology. The nanotube can be synthesised by the electrodeposition process [281] and the solution-phase [282] and hydrothermal [283] methods. The n-type Bi_2_Te_3y_Se_y_ and p-type Bi_2x_Sb_x_Te_3_ nanotube arrays shown in Figure 16a,b with an outer diameter of 200–220 nm and a wall thickness of 40–70 nm were successfully electrodeposited into the nanochannel of anodic alumina oxide (AAO) at room temperature using a potentiostat or galvanostatic method [284]. Bi_2_Te_3_ nanotubes with diameters smaller than 100 nm and spiral tube walls synthesised by a hydrothermal process demonstrated that adding nanotubes significantly lowers the thermal conductivity while having a minimal effect on the electrical conductivity, which in turn increases *ZT* of the Bi_2_Te_3_-based material [285].

Theoretical calculations predict that it is possible to achieve (PGEC) in zigzag graphene nanoribbons [286]. For example, a zigzag graphene nanoribbon with a 4 nm width and a 2 mm length could have a high *ZT* of 4 if it had extended line defects, impurities in the substrate, and rough edges [287]. Theoretical calculations show that a semiconducting single-walled nanotube (s-SWNT) with a smaller diameter can achieve a large power factor [234]. For example, MacLeod et al. (2017). showed enhanced power factor and reduced thermal conductivity of s-SWNTs with diameters of 1 nm that resulted in a *ZT* value of 0.12 at room temperature [288].

The composite of the nanotube with other materials can enhance the TE efficiency. For example, the SWCNT/PANI nanocomposites exhibit higher electrical conductivity and Seebeck coefficient than pure PANI, and the maximum power factor observed in the nanocomposites is 2 × 10^−5^ Wm^−1^K^−2^, which is more than a two-fold increase over pure PANI. As a result, a *ZT* of 0.004 is observed at room temperature [289]. The Seebeck coefficient of MWCNT/PANI nanocomposites (shown in Figure 16c,d) at room temperature is 22.4 μVK^−1^, which is about double that of a pure MWCNT sheet and eight times higher than that of pure PANI [290].

Choi et al. (2014) fabricated tellurium NW (TeNW) films hybridised with single-walled carbon nanotube (SWCNT) as a flexible TE material and demonstrated that this system exhibits high TE efficiency. The *ZT* of the system was 0.45 × 10^−2,^, which is higher than pure (TeNW) by one order [291]. Park et al. (2018) reported that (PbTe) nanotubes with an average diameter of 100 nm and a wall thickness of 20 nm (shown in Figure 16e,f) could be made on a large scale using a three-step process: electrospinning, electrodeposition, and cationic exchange reaction. They found that changing the ratio of Ag to Pb in the Ag_x_Te_y_-PbTe nanocomposites during the cationic exchange reaction made it possible to control their TE properties. When the amount of remaining Ag was 30%, the Seebeck coefficient reached its maximum of 433 μVK^−1^ (at 300 K), and the power factor reached its maximum of 0.567 μW m^−1^ K^−2^ for pure PbTe [292]. Table 2 explains and summarises the configurations of the 1D TE nanostructure with their characterization and properties.

### 4.2. The Effect of One-Dimensional Nanostructuring on the Enhancement of TE Generation Performance

#### 4.2.1. Thermal Conductivity

Reducing the diameter of 1D nanostructure, TE materials will increase phonon scattering and significantly reduce the thermal conductivity of the TE materials, which is the original purpose of making 1D nanostructure TE materials. For example, reducing the rough Si NW to 50 nm exhibits a 100-fold reduction in thermal conductivity compared with bulk Si [256]. Moreover, proper doping could be utilised to reduce thermal conductivity. By reducing the Si NWs diameter and tuning impurity doping levels, *ZT* values obtain a 100-fold improvement over bulk Si as a result of a huge reduction in thermal conductivity of 200 fold compared with bulk Si at 200 k that leads to *ZT* = 1 at the same temperature [300].

Heterostructures of 1D TE materials can be promising to reduce TE conductivity as it is an excellent tool to increase phonon scattering. For example, Ge–Si core–shell NWs exhibited a lower thermal conductivity than Ge NWs attributed to phonon scattering at the interface [301]. Nanotubes can be a very effective approach to reduce thermal conductivity. For example, silicon nanotubes have 33% lower thermal conductivity than silicon NWs [302].

The thermal conductivity of 1D TE material can be farther reduced by introducing porosity in the structure. For instance, single-crystalline porous silicon NWs exhibited extremely low thermal conductivity, as low as 0.33 Wm^−1^K^−1^ at 300 K and 43% porosity. This ultralow thermal conductivity is attributed to the reduction in phonon velocity caused by porosity [303].

#### 4.2.2. Seebeck Coefficient

Reducing the diameter of 1D TE materials could enhance the Seebeck coefficient of TE materials [304]. For example, the Seebeck coefficient of PbTe NWs with a diameter of 30 nm that were synthesised by the two-step hydrothermal method was raised to 628 μVK^−1^, which is about 137% higher than that of bulk PbTe [305]. Zhang et al. (2012) synthesised a Te–Bi2Te3 NWs heterostructure with a high Seebeck coefficient and low thermal conductivity [306]. Moreover, increasing the defects in the 1D TE materials could increase the Seebeck of the TE materials. For example, the vertically aligned porous silicon NWS arrays fabricated on the two sides of a silicon substrate using a simple metal-assisted chemical etching method exhibit a high Seebeck coefficient, up to 513 μV K^−1^, and low thermal conductivity, down to 1.68 Wm^−1^K^−1^ [307].

#### 4.2.3. Power Factor

Combining 1D TE materials with organic materials could enhance the power factor. For example, the addition of Bi_2_Te_3_ NWs to the conducting polymer P3HT exhibited an enhancement in the Seebeck coefficient without a significant effect on the electrical conductivity, which led to a large power factor of 13.6 μW m^−1^ K^−2^ at room temperature compared to that of 3.9 μW m^−1^ K^−2^ in P3HT. This enhancement of the TE performance is attributed to a possible energy-filtering effect at the P3HT–Bi_2_Te_3_ interface [308].

Zhou et al. (2017) fabricated flexible n-type TE single wall nanotube (SWNT) films that exhibit an ultrahigh power factor of 1500 μW m^−1^ K^−2^. The extensive power factor can be attributed to the high electrical conductivity of 3.63 × 10^5^ S m^−1^ and the Seebeck coefficient −64 μV K^−1^ [309]. Moreover, doping could increase the power factor and the performance of the 1d TE materials. For example, Avery et al. (2016) demonstrate that large TE power factors of semiconducting single-walled carbon nanotubes could be obtained by carefully controlling chirality distribution and carrier density while reducing the thermal conductivity possible by proper doping [310].

#### 4.2.4. Electrical Conductivity

Reducing the diameter of 1D TE materials might maintain or increase their electrical conductivity. This improvement in electrical conductivity is attributable to enhanced charge carrier mobilities because of DOS modifications [311]. Moreover, the electrical conductivity can be changed by changing the Fermi level, which leads to changing the concentration of the carriers. By using suitable dopants, the concentrations of carriers in these structures can be changed. For example, the electric conductivity of 1D nanostructure ZnO is shown to be higher than that of the bulk forms [312].

Surface roughness and impurities might decrease the electrical conductivity as it becomes an obstacle to carrier mobilities. For example, in their study, Curtin and Bowers (2014) discovered that gated NWs had much more excellent electrical conductivity than doped Si because of the lack of ionised impurities and increased carrier mobility [313]. Metallic surface states could be affected by the TE transport. For example, Shin et al. (2016) changed the surface-to-volume ratio (s/v) of Bi_2_Se_3_ NWs to study the effect of morphological surface states on TE transport and found the electrical conductivity of Bi_2_Se_3_ NWs increased as s/v increased [314].

### 4.3. Synthesis Methods

This section will discuss some of the most common and effective synthesis methods for producing large-scale 1D nanostructure TE materials. These methods play a crucial role in achieving the desired structural and compositional characteristics necessary for enhancing the TE performance of materials. The large-scale synthesis is critical to meet the demands of practical applications and enable the widespread implementation of TE technologies.

One widely used method for synthesizing 1D nanostructures is the vapour-phase deposition technique. This method involves the growth of nanowires, nanotubes, or nanorods through vapour-phase reactions, such as chemical vapour deposition (CVD) or physical vapour deposition (PVD). These techniques offer precise control over the growth parameters, allowing the synthesis of uniform and well-defined nanostructures. Moreover, tuning the reaction conditions and precursor composition bytuning the reaction conditions and precursor composition makes it possible to tailor the dimensions and properties of the resulting 1D nanostructures to optimise their TE performance.

Another commonly employed approach is the template-assisted synthesis method. This method utilises porous templates, such as anodised aluminium oxide (AAO) membranes or electrospun polymer fibres, as scaffolds for the growth of 1D nanostructures. By filling the template with the desired precursor materials and subsequently removing the template, highly ordered arrays of nanowires or nanofibers can be obtained. Template-assisted synthesis offers excellent control over the dimensions and alignment of the nanostructures, making it suitable for large-scale fabrication.

Solution-based methods, such as sol-gel, hydrothermal, or electrochemical deposition, are also widely utilised for synthesizing 1D nanostructure TE materials. These methods involve the formation of nanostructures through chemical reactions in solution or under specific conditions. Solution-based methods offer advantages such as low cost, simplicity, and scalability, making them attractive for large-scale synthesis. Additionally, these methods enable the incorporation of dopants or the formation of heterostructures that can further enhance the TE properties of the materials.

It is worth mentioning that advancements in bottom-up synthesis techniques, such as molecular self-assembly and colloidal synthesis, have also contributed to the production of large-scale 1D nanostructure TE materials. These methods utilise the self-organization of molecules or nanoparticles to form ordered nanostructures with controlled dimensions and properties. By carefully designing the precursor molecules or nanoparticles, it is possible to achieve tailored nanostructures with enhanced TE performance.

Overall, the choice of synthesis method depends on various factors, including the desired nanostructure morphology, composition, scalability, and compatibility with the chosen TE materials. The ability to produce large-scale 1D nanostructure TE materials through these synthesis methods is crucial for advancing the field and enabling their practical implementation in energy conversion and waste heat recovery systems. Further research and development in synthesis techniques will continue to drive progress towards efficient and cost-effective TE technologies. We have further discussed the general synthesis and fabrication processes required to achieve 1D-TE materials.

#### 4.3.1. Electrodeposition

**Figure 17:**
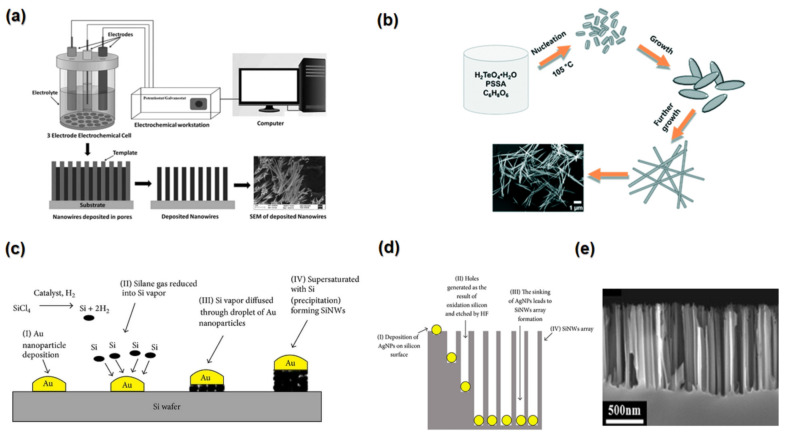
(**a**) Schematic steps for electrochemical deposition method “Reproduced with permission from [317], Elsevier, 2021”. (**b**) Schematic illustration of hydrothermal mechanism “Reproduced with permission from [329], Society of Chemistry, 2015”. (**c**) Schematic illustration of VLS mechanism of SiNWs growth “Reproduced with permission from [336], Hindawi, 2013”. (**d**) Electroless etching mechanism “Reproduced with permission from [336], Hindawi, 2013”. (**e**) The SEM images of the cross section of the silicon etched in the AgNO3/HF solution “Reproduced with permission from [338], Elsevier, 2011”.

Electrodeposition is an excellent method for synthesising nanostructured materials due to its low cost, fast deposition rate, large scale, and high adaptability for tailoring material characteristics [315]. Electrodeposition can make NWs from many different materials, like metals and semiconductors [316]. The electrodeposition mechanism starts with preparing the electrolyte solution by dissolving metal salts of the required material in an aqueous solution. Then, by applying a current or voltage between two conducting electrodes immersed in the electrolyte and connected with potential, the cathodic reduction of the dissolved metal cations produces film deposition on one of the electrodes. When a nanoporous template covers a cathode during electrodeposition, the deposition is restricted to the nanopores, allowing for the creation of NWs rather than thin films, as shown in Figure 17a [317].

Electrodeposition templates, such as anodic aluminium oxide (AAO) and porous polymer membranes, are often employed [318]. The AAO template is widely utilised because it has high pore density, and the pore sizes are easily adjustable [318]. The pore size of the AAO determines the diameter of NWs, and the length of the wire can be controlled by deposition time. The degree of pore-filling is influenced by nucleation rate, growth rate, and AAO template quality [319]. Using a template-assisted electrodeposition process, Bi_2_Te_3_ NWs with a diameter of less than 40 nm can be synthesised [320].

Three-electrode pulsed electrodeposition is one of the most used methods because of its flexibility, and precise control allows for producing highly oriented crystals with the uniform large-scale film [321,322]. Further, it is possible to produce NTs, multi-segment NWs, by electrodeposition [275,323]. Electrodeposition can be combined with lithography to produce NWs arrays [324]. Additionally, it is possible to produce NWs arrays using free template electrodeposition by depositing the seed layer before the electrodeposition growth [325]. Also, it is possible to produce NWs template-free and seed-free by two-step electrodepositions [326].

#### 4.3.2. Hydrothermal Synthesis

Hydrothermal synthesis is a flexible and cost-effective technique that uses solution ion reactions [327]. A Teflon-lined stainless-steel container combines all the necessary chemicals, including precursors, reductants, and surfactants [328]. The container is sealed tightly, heated to the reaction temperature, and kept at that temperature for the reaction time, then cooled to room temperature. The precursors may dissolve into ions in vessel at high temperatures and pressures. The ions may then react to form crystal nuclei, which is followed by a crystal growth process [327]. After the reaction, samples are filtered, washed, and vacuum dried.

Hydrothermal synthesis can produce crystals of different sizes (up to hundreds of micrometres) and shapes (NWs, nanorods, nanoplates, and micro belts). Reductants influence the nucleation rate, and the surfactants change the surface-adsorbed energy of certain crystal planes and cause the 1D nanostructures to form. For example, Park et al. (2015) demonstrated the hydrothermal synthesis of tellurium NWs (TeNWs), as shown in Figure 17b, with a diameter of 30–140 nm and a length of several micrometres, at 105 °C and using telluric acid as a tellurium source, ascorbic acid as a weak reducing agent, and a linear polymer as a template. The NWs have a high Seebeck coefficient and power factor of about 568 μVK^−1^ and 8.44 μW m^−1^ K^−2^, respectively [329]. As another example, n-type Bi_2_Te_3_ NWs synthesised by hydrothermal synthesis demonstrated an increased *ZT* of 0.96 at 380 K [330].

#### 4.3.3. Vapor Liquid Solid Growth

Numerous successful semiconductor nanowire syntheses have been made using the vapour-liquid-solid (VLS) growth method. This process was initially established to synthesise silicon NWs with different diameter ranges [331]. To start the VLS growth process, metal catalyst nanoparticles like (Au, Fe, Pt, Al, etc.) were first deposited onto the silicon (Si) wafer substrate [332]. Silicon wafer coated with metal catalysts was then heated to above its eutectic point in a chemical vapour deposition system [333]. Then, the silicon gas, such as silane (SiCl_4_) or tetrachlorosilane (SiH_4_), was introduced into the reaction tube and turned into silicon vapour and decomposed through a metal catalyst to make droplets of a metal-silicon alloy [333]. Once the liquid alloy was oversaturated, nucleation and growth occurred when the catalyst droplet met the substrate [334]. This seed acts as a favourable location for material to be deposited, helps the seed grow into an NW, and stops other nucleation from occurring on the same catalyst [334]. The diameters of the NWs were determined by the size of the produced alloy droplet, and their lengths were determined by the reaction duration [335]. Figure 17c illustrates the mechanism of NW’s growth by VLS [336]. Vertical-aligned SiNWs array can be synthesised by employing the AAO template [335].

#### 4.3.4. Electroless Etching Method

As a low-cost, simple, low-temperature process, electroless etching (EE) can be used to fabricate SiNWs arrays [337]. This process is quick and requires sophisticated equipment (2–30 min) [338]. High-density pores and rough surface NWs could be created by employing electrochemical procedures, which is impossible with conventional techniques [256]. This procedure includes two primary steps: electroless metal (silver, nickel, platinum, and gold) deposition on a silicon wafer and chemical etching in a fluoride-ion-based solution [339]. When a cleaned Si wafer is immersed in an NH_4_HF_2_ and AgNO_3_ solution, silver ions ill draw electrons from the silicon substrate due to the deposition of silver nanoparticles on the silicon surface [340]. The holes develop as the silicon beneath the Ag nanoparticle is oxidised and subsequently etched by the HF etchant [338]. The created holes act as a sinking path for the leftover Ag nanoparticles, developing SiNWs arrangements via a longitudinal and lateral silicon suspension [340]. Figure 17d,e illustrates the mechanism of electroless etching (EE) [338].

### 4.4. Performances of Characterizing Thermoelectric Nanowires

According to Equation (1), to calculate the value of the *ZT* of materials, measuring the Seebeck coefficient, electrical conductivity, and thermal conductivity is necessary. TE characteristics may be measured using several different techniques. However, several factors must be considered before choosing the proper method, including the material’s type, dimensions, and forms; the desired temperature range; and environmental stability. There are three categories of measurement techniques: NWs array thin film; bulk NWs nanocomposites; and individual NWs.

The electrical conductivity of a material can be determined through commonly employed techniques, such as the two-point probe method, the four-point probe method, and van der Pauw’s method. In the two-point probe method, a copper wire is connected to both ends of the specimen. By passing a known current through the sample, the resulting voltage drop across the wire can be utilised to measure the material’s resistivity. Although the two-point probe method is straightforward, it is prone to errors arising from various sources, including the inclusion of contact resistance, probe resistance, and spreading resistances beneath the probes, which contribute to the overall circuit resistance [341].

**Figure 18:**
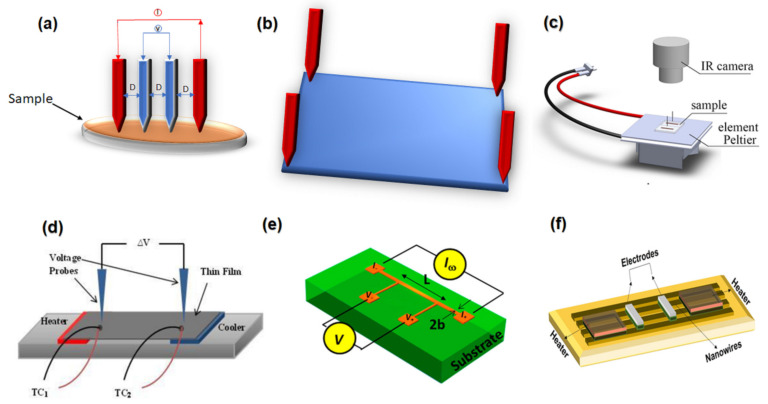
(**a**) Schematic illustrating the four-probes electrical conductivity measuring methods. (**b**) Schematic illustrating van der Pauw’s electrical conductivity measuring methods. (**c**) Schematic illustrating the Seebeck measuring device of the vertical TE thin film generator “Reproduced with permission from [343], Elsevier, 2019”. (**d**) Schematic illustrating in-plane Seebeck measuring device for TE thin film generator “Reproduced with permission from [347], MDPI, 2021”. (**e**) Schematic illustrating of the 3ω method for measuring thermal conductivity for thin films “Reproduced with permission from [344], AIP, 2018”. (**f**) Image of microchip device used to measure of the TE performance of individual NWs accurately.

The four-point probe method is one of the best and most common ways to determine the resistivity of thin films of semiconductors. The four-point probe comprises four electrodes spaced at a distance D evenly in a linear array, as shown in Figure 18a. When the current (I) flows through the two outer electrodes, the voltage (V) will be measured across the inner electrodes. This approach measures the resistivity of thin film specimens with varied shapes. When testing with four probes, some considerations should be made, such as the uniformity of the film, whether it contains any isolated holes, the probes being at the same temperature, and the Seebeck voltage, so it is preferable to use an alternative current to ignore the voltage [342]. In van der Pauw’s method, the contacts are not in a line on the sample’s surface like in the four-point method. Instead, they are around the edge of the sample, as shown in Figure 18b. However, the sample should be uniform, symmetrical, and not have isolated holes [342].

The Seebeck coefficient may be determined using a heater and a heatsink to generate a temperature differential across a TE material and two thermocouples to measure the resulting voltage drop. The measuring point of the thermocouple could be on the heater and the sink and sometimes on the sample directly [341]. To measure the Seebeck of the thin film TEGs, the sample is placed on a heater and the voltage differences between n-type and p-type material are measured by nanovoltmeter; the temperature can be measured by thermocouples or IR camera, as illustrated in Figure 18c [343]. Finally, it is simple to measure the in-plane Seebeck of thin film TE materials by placing the sides of the thin film on cold and hot sources, then measuring the voltage difference by nanovoltmeter between the hot film side and the cold film side; the temperature can be measured through a thermocouple, as shown in Figure 18d [344].

A variety of thermal conductivity measuring methods belong to the two categories of the study state and dynamic state over all these methods. The laser flash method and 3ω method are the most common measurement methods for thermal conductivity in TE materials [345]. The laser flash method is employed as a versatile technique for determining the thermal properties of glasses, metals, and ceramics, offering notable advantages and few limitations. In this method, a brief, intense pulse of heat is directed towards a specific region of the sample, while an infrared (IR) detector placed on the opposite side monitors the heat dissipation rate over a given period. This non-destructive approach enables accurate thermal conductivity and diffusivity characterisation in a wide range of materials.

The 3ω method is commonly used to measure thermal conductivity for thin films and solid materials. A metal strip is deposited onto the film surface. An AC current with a frequency ω (I_ω_) of angular modulation is passed through the metal, which works as a heater and a thermometer, causing the temperature to fluctuate with a frequency of 2ω, which creates a higher frequency voltage of 3ω. The higher harmonics are detected with amplifiers, which are used to determine thermal diffusivity, and from this, we can calculate thermal conductivity, as shown in Figure 18e [345].

Acquiring accurate values of these parameters is challenging because of errors caused by heat loss and radiation from NWs surfaces, wires, or plates connected with the sample during the measurement. Moreover, the instability of the material caused by the high temperature during testing leads to the decomposition of the material, then mischaracterization and oxidation of the material, which leads to reduced heat and electric transfer [346].

Microchip devices and scanning probe microscopy (SPM) are the most common methods for characterizing a single wire. Microchip devices, where the NWs are grown or transferred onto the microchip, are widely used to accurately measure TE conductivity, electric conductivity, and Seebeck coefficient for single NW TE materials with minimal diameters, as shown in Figure 18f. Unlike microchip devices, SPM has a competitive advantage over the microchip because it is a non-destructive test that measures transport properties of a single NW both within and outside a matrix using different probes; it also prevents the sample oxidation that may occur during microchip device fabrication. However, to measure the properties of the NWs, the diameter of the probe must be smaller than the diameter of the NWs [346].

## 5. Summary and Future Scope

The growing demand for clean energy and pollution reduction underscores the significance of TE technology. TE has the potential to harness waste heat from conventional and renewable energy sources. Despite scientific endeavours dating back to the 18th century, when the direct conversion of heat to electricity was first observed, achieving high efficiency and low-cost, large-scale fabrication using eco-friendly materials remains a challenge.

A diverse range of TE materials is available, characterised by their abundance, non-toxicity, and cost-effectiveness. However, selecting the appropriate material depends on the specific application, temperature range, and working conditions. To effectively utilise chosen TE materials, enhancing their performance while concurrently developing fabrication and application methods is crucial. Enhancing the performance of TE materials is a complex task due to the interdependent nature of their parameters. Various methods, with a particular focus on nanostructuring, can be employed to improve TE efficiency.

Among the influential and favourable nanostructured materials, 1D nanostructures demonstrate significant potential for diverse applications and can be fabricated using different techniques. Examples include quantum wires, nanowires (NWs), nanorods, nanofibers, nanotubes, and heterostructures. Each type possesses unique properties that can be leveraged to enhance TE performance or application. For instance, nanowires exhibit a remarkable Seebeck effect, heterostructures effectively reduce thermal conductivity, and fibres are suitable for wearable TE devices. Combining the 1D nanostructuring approach with other development methods, such as proper element doping in nanowires, can further enhance TE performance.

While numerous fabrication methods exist, selecting the appropriate one is crucial, as it can significantly impact the performance and complexity of TEGs and the associated cost. Therefore, careful consideration must be given to ing a fabrication method that meets cost-effectiveness and large-scale synthesis without compromising performance. Additionally, accurate electrical conductivity, thermal conductivity, and Seebeck coefficient measurements are essential for assessing TE material performance. Various measurement techniques are available, and the selection depends on the material type, size, form, and operating temperature range. Emphasis should be placed on choosing abundant earth and eco-friendly TE materials and enhancing their parameters through a combination of methods, including 1D nanostructuring, doping, band engineering, and other configurations.

Furthermore, efforts should be directed towards developing large-scale synthesis methods for high-performance TE materials at a low cost. Finally, there is a need to develop performance characterization methods that are cost-effective, simple, and accurate and are specifically tailored for unique 1D nanostructured TE materials. The future scope of TE materials research holds great significance and potential for various areas of science and technology. Here are some key aspects and their importance:

- Energy Conversion and Harvesting: TE materials can convert waste heat into useful electrical energy, providing a pathway for enhanced energy efficiency and sustainability. Further research can lead to developing high-performance TE materials that can efficiently harness waste heat from industrial processes, automotive systems, and renewable energy sources.
- Renewable Energy Integration: As the world moves towards a clean energy future, TE materials can be crucial in integrating renewable energy sources. By converting heat energy from concentrated solar power, geothermal sources, and biomass, TE devices can contribute to renewable energy systems’ overall efficiency and stability.
- Portable and Wearable Electronics: The miniaturization and increasing complexity of electronic devices pose challenges in terms of power supply. TE materials can enable the development of portable and wearable electronics that utilise body heat or ambient temperature variations to generate electricity, eliminating the need for conventional batteries and enhancing the device’s energy autonomy.
- Space Exploration and Remote Sensing: In space missions and remote sensing applications, where power sources are limited and inaccessible, TE materials can provide a reliable and efficient electricity generation. Further advancements in TE materials can significantly enhance the performance and reliability of power systems for space exploration and remote sensing technologies.

Overall, continued research and development in TE materials are important in addressing global energy challenges, promoting sustainability, and enabling technological advancements in various fields. With advancements in material synthesis, device engineering, and characterization techniques, the future of TE materials research looks promising for unlocking their full potential to create a greener and more energy-efficient world.

## Figures and Tables

**Table 1:** Summaries the most well-known TE materials depend on their working temperature, their TE performance, and their properties.

No	State of Art Material	Material	Conductivity Type	*ZT*	Working Temperature (K)	Seebeck Coefficient (μVK^−1^)	Thermal Conductivity (Wm^−1^K^−1^)	Electrical Conductivity (S.cm^−1^)	Stability	Toxic Level	Cost	Ref.
1	Bi_2_Te_3_	Bi_0.5_Sb_1.5_Te_3_	p-type	1.86	320	242	0.65	650				[165]
		Te-embedded Bi_2_Te_3_	n-type	2.27	375	−234	0.80	825				[166]
2	Ternary Composites	SWNT/PANI	p-type	0.12	300	65	0.43	769				[85]
		CNTs/PEDOT	n-type	0.5	300	−1234	0.67	6.9				[167]
3	PbTe	Pb_0.98_Na_0.02_Te–8%SrTe	p-type	2.5	923	300	0.9	250				[58]
		PbTe_0.996_S_0.001_I_0.003_	n-type	1.7	800	−225	1.25	500				[168]
4	SnSe	SnSe	p-type	2.6	923	350	0.35	100				[65]
		SnSe_0.97_Br_0.03_	n-type	2.8	773	−480	0.25	41				[169]
5	Perovskite	LaCo_0.92_Ni_0.08_O_2.9_	p-type	0.2	300	220	0.35	33.3				[170]
		La-doped SrTiO_3_	n-type	0.27	1073	−300	3.1	80				[111]
6	Skutterudites	Ce_0.6_Ni_1.5_Co_2.5_Sb_12_	p-type	0.45	850	153	1.62	600				[171]
		Ba_0.3_In_0.3_Co_4_Sb_12_	n-type	1.4	850	−190	2.5	1300				[172]
7	Zintl Phase	YbCd_1.85_Mn_0.15_Sb_2_	p-type	1.14	650	250	0.4	200				[173]
		Mg_3.15_Mn_0.05_Sb_1.5_Bi_0.49_Te_0.01_	n-type	1.85	723	−310	0.8	220				[174]
8	clathrates	Ba_8_Au_5.3_Ge_40.7_	p-type	0.9	680	150	1	50				[175]
		Ba_8_Ni_0.31_Zn_0.52_Ga_13.06_Ge_32.2_	n-type	1.2	1000	−200	1.5	400				[102]
9	Metal sulphides	CU_1.97_S	p-type	1.7	1000	300	0.5	100				[123]
		AgBiS_2_	n-type	0.7	820	−300	0.5	120				[176]
10	Half-Heusler	(Nb_0.6_Ta_0.4_)_0.8_Ti_0.2_	p-type	1.6	1200	220	1.5	550				[177]
		Zr_0.2_Hf_0.8_NiSn_0.985_Sb_0.015_	n-type	1.1	1000	−190	5.5	1500				[178]
11	Oxides	Ca_3_Co_4_O	p-type	0.29	1073	155	4.4 (at room temp)	151				[179]
		ZnO	n-type	0.44	1000	−300	2	100				[114]
12	Si-Ge alloys	Si_0.62_Ge_0.32_Ni_0.03_B_0.03_	p-type	1.56	1000	321	1.47	250				[180]
		SiGe–Mg_2_Si	n-type	1.3	950	−340	2.7	240				[79]

Green: favourable; Orange: neutral; Red: Unfavourable.

**Table 2:** Different types of 1d nanostructured configurations with their synthesis method and TE characterization.

1D Nanostructure Configuration	Synthesis Method	Description	Seebeck Coefficient (µV/K)	Electrical Conductivity (S/cm)	Thermal Conductivity (W/m·K)	Power Factor (μW/K^2^.m)	*ZT*	Ref.
Quantum wires	theoretically	0.5 nm diameter of Bi_2_Te_3_	-	-	-	-	14	[28]
	EBD synthesis method	1D Te QWs formed locally in the aluminium oxide layer	−800 at 300 K	-	-	-	-	[293]
Nanowires and nanorods	Theoretically	Bi NW	-	-	-	-	6 at 77 K	[245]
	Electrochemical Deposition, AAO template	Bi_0.5_Sb_1.5_Te_3_ NWs with 67 nm diameter	−150	480	-	1080	-	[294]
	Solution-grown	Bi_2_Te_3_ nanorods with 40 nm diameter	−180	-	-	-	-	[295]
Nanofibers	powder-processing	Bi_0.5_Sb_1.5_Te_3_ fibres	−(207–268)	153–189	-	0.3–0.4	-	[296]
Heterostructures	bench top solution method	Bi_2_Te_3_/Sb_2_Te_3_ core-shell	−110	-	0.4	-	0.4 at 480 K	[297]
	solution-phase	PbTe/Bi_2_Te_3_ segments NWs achieve	−300	110	0.51	1000	1.2 at 600 K	[298]
Nanotubes	hydrothermal synthesis	Bi_2_Te_3_ nanotube	-	-	0.3	-	-	[285]
	bottom-up synthesis and SPS	Au-doped Bi_2_Te_3_ nanotube	-	-	0.47	2300	0.95 at 480 K	[299]

## Data Availability

Not applicable.

## Abbreviations

Symbols	
$C_{v}$	Specific Heat at constant volume
$C_{P}$	The specific heat
$D_{T}$	Thermal diffusivity
e	Carrier charge
I	Current
Isc	Short circuit current
E	Electron energy
$E_{f}$	Electron energy at the Fermi level
$g\left(E\right)$	The density of states
$f\left(E\right)$	Fermi function
h	Planck’s constant
k	Thermal conductivity (W/m·K)
$k_{e}$	Electron thermal conductivity
$k_{l}$	Lattice thermal conductivity
$K_{B}$	Boltzmann constant
L	Lorenz number
l	Phonons mean free path
𝑛	Carrier density
$N_{v}$	Valley degeneracy
𝑁𝑚*	Effective mass
m𝑣b*	Band-mass
QH	Thermal energy (hot side) (J)
S	Seebeck coefficient
T	Temperature (K)
T_c_	The temperature on the cold side
T_h_	The temperature on the hot side
V	Voltage (V)
Voc	The open circuit voltage
W	Power generated (W)
v	Average Sound velocity
σ	Electrical conductivity (S/m)
$\sigma \left(E\right)$	Electronic conductivity specified in terms of the Fermi energy
η	Energy conversion efficiency
μ	Carrier mobility
ρ	Mass density
Abbreviations	
AAO	anodic aluminium oxide
CNT	Carbon nanotube
MWCNT	Multi-wall carbon nanotube
NWs	Nanowires
PANI	Polyaniline
PEDOT: PSS	Poly(3,4-ethylenedioxythiophene): poly (styrene sulfonate)
PDLC	Polymer-dispersed liquid crystals
PGEC	Phonon glass electron crystal
PTENG	Photo-Thermoelectric Nanogenerator
rGO	Reduced graphene oxide
TCOs	Transparent conductive oxides
TCM	Thermochromic materials
TC	Transition temperature
TE	Thermoelectric
TEGs	Thermoelectric generators
TENG	Thermoelectric nanogenerator
SWCNT	Single-wall carbon nanotube
ZT	Figure of merit
1D	One-dimensional
